# The relationship between human blood metabolites and preeclampsia-eclampsia: A Mendelian randomization study

**DOI:** 10.1097/MD.0000000000037505

**Published:** 2024-03-29

**Authors:** Jiping Wei, Liyuan Huang, Mingda Wu, Xiaodan Lu, Yongfu Song, Yongji Wang, Yan Guo

**Affiliations:** a School of Clinical Medicine, Changchun University of Chinese Medicine, Changchun, China; b Precision Medical Center, Jilin Province General Hospital, Changchun, China; c Department of Pediatrics, The Affiliated Hospital to Changchun University of Chinese Medicine, Changchun, Jilin, China.

**Keywords:** causal inference, genetic variation, Mendelian randomized study, preeclampsia and eclampsia, serum metabolites

## Abstract

Preeclampsia and eclampsia are serious complications of pregnancy, leading to high rates of maternal and neonatal mortality. During pregnancy, there are changes in relevant serum metabolites in women. However, it remains unclear if these serum metabolites contribute to the development of associated disorders during pregnancy. Therefore, we conducted a Mendelian randomization study to explore the causal relationship between serum metabolites and preeclampsia and eclampsia. We utilized the inverse variance weighted model as our primary analysis approach. We complemented this with sensitivity analyses, including the heterogeneity test, horizontal pleiotropy test, and leave-one-out analysis, to ensure the robustness of our findings. Furthermore, we conducted linkage disequilibrium score regression, multivariable Mendelian randomization, and metabolic pathway analysis to further explore the genetic data. The Mendelian randomization analysis has identified γ-glutamylglutamine, inosine, and isoleucine 10 metabolites that are significantly associated with preeclampsia, and γ-glutamylglutamine and phenylacetate 8 metabolites that may potentially contribute to the development of eclampsia. Notably, γ-glutamylglutamine has been found to have a causal relationship with both preeclampsia and eclampsia. In the multivariable Mendelian randomization analysis, our research findings suggest that both isoleucine and X-14304–leucylalanine directly impact preeclampsia within the context of amino acids and peptides. Moreover, our observations reveal that carbohydrates can also have a direct effect on preeclampsia. Importantly, it should be emphasized that only 3-lactate in amino acids has been shown to have a direct influence on eclampsia. This research has the potential to enhance our understanding of the biological variances related to disease status, providing a foundation for future investigations.

## 1. Introduction

Preeclampsia and eclampsia (PE) are substantial complications than can arise during pregnancy, leading to maternal and newborn mortality worldwide, affecting 3%–8% of pregnant women.^[1–4]^ PE increases the risk of adverse pregnancy outcomes, such as preterm birth and low birth weight,^[5,6]^ as well as posing serious health risks for both mother and infant including chronic hypertension, myocardial ischemia, end-stage renal disease during pregnancy,^[6,7]^ respiratory disorders, and cognitive impairment in newborns.^[7,8]^ The exact cause of PE remains unknown. Previous research has elucidated several mechanisms underlying these complications. These mechanisms include impaired remodeling of maternal uteroplacental spiral arteries due to acute atherosclerosis,^[9]^ the decrease in vascular endothelial growth factor and placental growth factor, the rise in placental soluble fms-like tyrosine kinase 1,^[10]^ placental oxidative stress,^[11]^ and immune dysregulation.^[12]^ It is worth noting that PE specifically affects pregnant women and can be managed following childbirth.^[1]^

Serum metabolites play a crucial role in maintaining the health of both the mother and fetus, and significant alterations have been observed in the blood of women.^[13]^ These small compounds are found in human cells, organs, and bodily fluids, serving as substrates and products of energy metabolism and also originating from microbes and xenobiotics in the body.^[14]^ Alterations in metabolite concentrations can serve as valuable biomarkers for pathological diagnosis and prognosis.^[15]^ By detecting subtle changes in body metabolites, it becomes possible to gain a comprehensive understanding of physiological states, pathological processes, and the underlying mechanisms of complex diseases.^[14]^ However, despite extensive efforts in metabolite identification and validation, individual differences influenced by genetic and environmental factors remain challenging to overcome.^[16,17]^ Controlling for these confounding factors in observational studies is difficult, limiting the ability to establish causal inferences between serum metabolites and PE.

Mendelian randomization (MR) offers a fresh perspective for exploring the causal connection between serum metabolites and PE. By drawing on summary data from genome-wide association studies (GWAS), MR offers a broadly applicable method for evaluating the causal effects of an exposure on an outcome, using genetic variants as instrumental variables (IVs). To maintain the integrity of the MR study design, it is crucial that the genetic variants, which serve as proxies for the biological impact of a modifiable exposure, are causally associated with the corresponding disease risk. This approach is less prone to bias compared to observational studies, primarily because environmental factors are deemed unlikely to influence genetic variation.

## 2. Materials and methods

### 2.1. Study design

**Figure 1.**
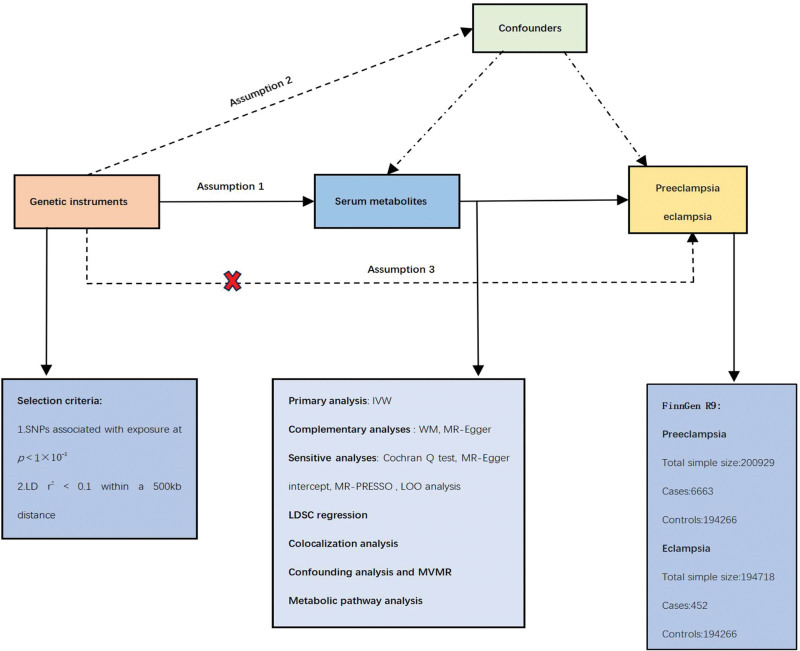
The overview of the research workflow. IVW = inverse variance weighted, LD = linkage disequilibrium, LOO = leave-one-out, MR-PRESSO = MR-Pleiotropy RESidual Sum and Outlier, MVMR = multivariate Mendelian randomization, SNPs = single nucleotide polymorphism, WM = weighted median.

The publicly available dataset used in this study has been ethically approved and can be accessed on the database website. To ensure the validity of the MR study, 3 key assumptions need to be met: the IVs employed are highly correlated with the exposures under scrutiny; the IVs are devoid of any confounding factors; the IVs do not have a direct effect on the outcome but solely influence the outcome via the exposures.^[18]^ In this investigation, we employed MR analysis by utilizing data from a GWAS to investigate the link between 486 blood metabolites (exposure) and the incidence of PE (outcome) in the Finnish populace. Figure 1 presents a graphic depiction of our study design. Our study design was influenced by the MR inquiry conducted by Cai et al.^[19]^

### 2.2. GWAS data for human blood metabolites

We obtained genetic data for blood metabolites from the metabolites GWAS server (http://metabolomics.helmholtz-muenchen.de/gwas/). The study conducted by Shin et al^[17]^ represents the most extensive investigation to date into the genetic loci associated with blood metabolites. With the aim of establishing a link between genetic variation and blood metabolism, Shin et al^[17]^ conducted a rigorous analysis, sequencing the complete genomes of 7824 adults from the Twins UK and Kora databases in Europe. To ensure accuracy and efficiency, the researchers employed various techniques to refine the acquired data. By performing high-throughput metabolic profiling through genome-wide association scans, they identified approximately 2.1 million single nucleotide polymorphisms (SNPs) associated with 486 metabolites and human genetic variants.^[17]^ Based on data from the Kyoto Encyclopedia of Genes and Genomes database, a total of 309 known metabolites have been categorized into 8 classes: cofactors and vitamins, energy, amino acid, carbohydrate, lipid, nucleotide, peptide, and xenobiotic metabolism.^[20]^ The experiment made use of liquid chromatography, gas chromatography, and a combination of *t* and mass spectrometry methodologies. After completing the quality control process, correlations were established between the concentrations of all 486 metabolites in the Twins UK and Kora datasets and each SNP. This correlation analysis was based on the haplotype map input genotype dataset and conducted separately using linear regression models from Merlin and Quick Test of Tware. Subsequently, a genetic correlation analysis was conducted for the 486 blood metabolites obtained from 486 individuals, following the quality control procedures.

### 2.3. GWAS data for PE

GWAS summary statistics for PE were obtained from the R9 release data of the Finland consortium. The phenotype of interest in this study was “preeclampsia and eclampsia.” The preeclampsia GWAS included a total of 200,929 Finnish adult female subjects, with 6663 cases and 194,266 controls. The eclampsia GWAS included 194,718 Finnish adult female subjects, with 452 cases and 194,266 controls (Supplementary Table S1, Supplemental Digital Content, http://links.lww.com/MD/L931). During the course of the analysis, we carefully considered potential confounding factors, including but not limited to, sex, age, the first 10 principal components, and the genotyping batch. To ensure the robustness and credibility of the outcomes, meticulous adjustments were implemented to account for these variables, thereby preserving the integrity and enhancing the accuracy of the findings.

### 2.4. Selection of IVs

Initially, unitary criteria were applied to select SNPs from the 486 serum metabolites. In order to establish a rigorous threshold for significance, we meticulously set the cutoff at 1.00E-5 (*P* < 1 × 10^−5^), thereby ensuring that only the most highly significant results were included for analysis. Furthermore, to uphold genomic independence and minimize confounding factors, we strictly enforced a requirement for a linkage disequilibrium R2 value below 0.1 within a 500 KB range. By implementing these rigorous parameters, our goal was to optimize the reliability and precision of our analyses, thereby enhancing the validity of the obtained results. Moreover, we assessed the predictive power of these IVs in determining causal effects through 2 key parameters: the explained genetic variation (R2) and the F-statistic. Typically, an F-statistic >10 is used as a threshold to identify strong IVs. Any metabolite with an F-statistic of <10 will be excluded. Furthermore, we specifically extracted the SNPs that were linked to the specific exposure of our interest from the outcome. We applied a rigorous exclusion criterion, eliminating any SNPs that showed a significant association with the outcome (*P* < 1 × 10^−5^). This careful selection process aimed to ensure that only the relevant SNPs were included in the subsequent analyses, minimizing any potential confounding effects. Finally, we conducted MR analysis on metabolites that had more than 2 SNPs.

### 2.5. Statistical analysis and sensitivity analysis

Considering the fundamental assumption of SNPs validity, it was determined that the random-effect inverse variance weighted (IVW) method offered the utmost precision and accuracy in estimation. Consequently, our focus was primarily on evaluating the causal relationship between blood metabolites and PE, utilizing the findings obtained from the random-effect IVW method. This approach ensured that any potential confounding factors and biases were appropriately accounted for, enabling us to draw robust conclusions regarding the causal effects under investigation. We primarily used the IVW method with a *P*-value threshold of <.05 to assess causality between blood metabolites and PE, given its ability to yield robust causal inferences.^[21]^ This method, first proposed by Burgess et al,^[22]^ is widely used in MR studies. In addition, we performed complementary analyses using the MR-Egger and weighted median (WM) methods, which provide more robust estimates under less strict assumptions. The WM method can accommodate up to 50% invalid SNPs, while the MR-Egger method can detect horizontal pleiotropy and heterogeneity in the presence of such pleiotropy for all SNPs.^[21,23]^ As a comprehensive part of our sensitivity analysis, we employed four supplementary methodologies to further strengthen the robustness of our findings. These methods include the Cochran-*Q* test, MR-Egger intercept, leave-one-out analysis (LOO), and MR-Pleiotropy RESidual Sum and Outlier (MR-PRESSO). By utilizing these additional approaches, we were able to thoroughly evaluate the potential impact of outliers, heterogeneity, and directional pleiotropy, thereby enhancing the reliability and validity of our results. This comprehensive sensitivity analysis allowed us to address potential limitations and ensure the accuracy of our conclusions. A *P*-value below .05 obtained from the Cochran-*Q* test indicated heterogeneity in the results.^[24]^ Horizontal pleiotropy was evaluated using the MR-Egger intercept.^[25]^ To assess the influence of individual SNPs on the MR estimates, a LOO analysis was conducted.^[23]^ In summary, we conducted a thorough screening of blood metabolites to identify potential causal effects on PE. We employed multiple criteria, including significant *P*-values from the primary analysis (IVW-derived *P* < .05), consistent direction and magnitude across 3 MR methods, absence of heterogeneity or horizontal pleiotropy in the MR results, and minimal disruption of MR estimates by a single SNP. Additionally, we calculated the statistical power of our estimates using an online tool (https://shiny.cnsgenomics.com/mRnd/)^[26]^ to assess their reliability. This tool employs asymptotic theory to calculate power values for detecting causal effects obtained from IVs. With a type I error rate of .05, we determined power by considering the R2 of IVs, the proportion of cases with an outcome, and the odds ratio derived from the IVW analysis.

### 2.6. Genetic correlation and direction validation

Previous research indicates that genetic correlations between traits can lead to false positive MR results.^[27]^ While excluding SNPs related to PE during the IV selection process, the potential for unrelated SNPs to influence the genetics of PE cannot be completely ruled out. As a result, we proceeded to assess the genetic association between the identified metabolites and PE by employing linkage disequilibrium score regression (LDSC). This approach allowed us to investigate whether there was a disrupted causal effect due to shared genetic architecture. LDSC regression employs Chi-squared statistics based on SNPs to estimate the coinheritance between 2 traits.

### 2.7. Confounding analysis and multivariable MR analysis

In order to evaluate the horizontal pleiotropy of our MR findings and identify any SNPs that may have violated the MR assumptions, we performed a comprehensive set of sensitivity analyses. However, it is crucial to recognize that the potential for residual confounding due to unmeasured or unaccounted SNPs still exists. To tackle this issue, we employed the PhenoScanner V2 (http://www.phenoscanner.medschl.cam.ac.uk) website to assess the potential connection between each SNP and established risk factors for PE. These risk factors encompass high body mass index, elevated fasting blood glucose levels, high cholesterol levels, elevated triglyceride levels, as well as inadequate amounts of vitamin A and vitamin C. In the event that any SNPs were discovered to be correlated with the confounding factors mentioned above (*P* < 1 × 10^−5^), an additional MR analysis would be conducted, excluding these particular SNPs. This step is crucial in guaranteeing the validity and accuracy of our findings within the field. By ensuring that genetic variants are unequivocally associated with a singular risk factor during the MR analysis, we can establish robust and reliable results. However, it is worth noting that certain genetic variants may be associated with multiple risk factors, a phenomenon known as pleiotropy. To address this concern, we implemented the multivariable MR (MVMR) approach, a methodology that takes into account the potential interactions between multiple exposures, thereby rectifying any influence of genetic variations on these interactions. By incorporating this approach, we were able to accurately correct for and consider the complex interplay between various factors, resulting in a more comprehensive analysis. MVMR was performed using IVW^[28]^ and MR-PRESSO.^[29]^ In the MVMR analysis, we employed the instrumental variable weighted (IVW) method, which encompasses regressing all relevant exposed SNPs against the outcome of interest. These SNPs were then weighted according to the inverse variance of the outcome. By utilizing this robust statistical approach, we were able to effectively quantify the associations between the genetic variants and the outcome, while accounting for potential confounding factors and maintaining the integrity of the analysis. MR-PRESSO, on the other hand, can detect and remove outliers to address the pleiotropy issue associated with the IVs.

### 2.8. Metabolic pathway analysis

To gain a deeper understanding of the specific biological mechanisms that underlie the causal effects of blood metabolites on PE, we conducted a comprehensive metabolic pathway analysis. This analysis aimed to provide insights into the intricate pathways and interactions that contribute to the observed associations. This analysis involved utilizing the Kyoto Encyclopedia of Genes and Genomes database and the Metabo Analyst 5.0 tool (https://www.metaboanalyst.ca/) to examine the metabolic pathways associated with the known metabolites.

## 3. Results

### 3.1. Primary analysis and sensitivity analysis

**Figure 2.**
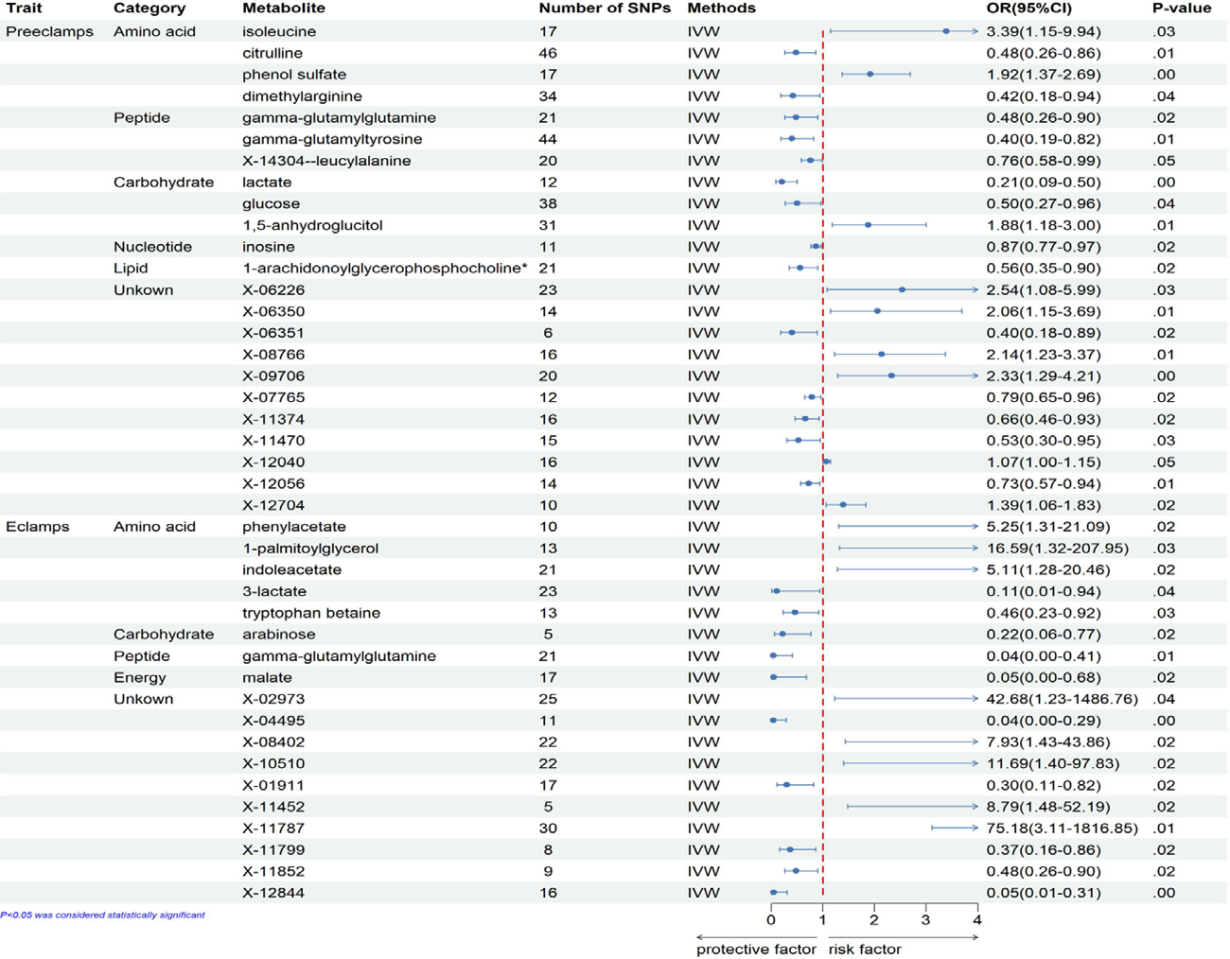
Forest plot for the causality of blood metabolites on preeclamps and eclamps derived from IVW analysis. CI = confidence interval, IVW = inverse variance weighted, OR = odds ratio, SNPs = single nucleotide polymorphisms.

We conducted MR studies on a total of 486 blood metabolites, adhering to rigorous procedures for instrument selection. Due to an insufficient number of SNPs for one of the serum metabolites, we carried out a total of 485 MR analyses. The F-statistics for all the SNPs associated with the metabolites exceeded 10, indicating a high power of the IVs. Supplementary Table S2, Supplemental Digital Content, http://links.lww.com/MD/L932, displays the F-statistic for all SNPs. The IVW analysis initially identified 23 metabolites significantly associated with preeclampsia, including 12 metabolites with known chemical properties and 11 metabolites with unknown chemical properties, as well as 18 metabolites with potential eclampsia causality, 10 of which contained unidentified chemical components (Fig. 2; Supplementary Table S3, Supplemental Digital Content, http://links.lww.com/MD/L933). Chemically, metabolites are categorized as amino acids, carbohydrates, peptides, nucleotides, lipids, and energy. Following a combination of complementation and sensitivity studies, 10 suitable metabolites fulfilling stringent screening requirements were found as candidate metabolites for preeclampsia, and 8 metabolites satisfied the criterion for acceptable candidate metabolites related with the risk of eclampsia (Table 1), including isoleucine, citrulline, phenol sulfate, dimethylarginine, γ-glutamylglutamine, leucylalanine, lactate, glucose, inosine, 1-arachidonoyl-glycerophosphocholine, phenylacetate,1-palmitoylglycerol, indoleacetate, 3-lactate, tryptophan betaine, arabinose, γ-glutamylglutamine, malate. The robustness of the causal relationship was confirmed through MR estimations, specifically with the use of WM and MR-Egger methods. These estimations consistently showed the same direction and magnitude of effect (Supplementary Figure 2, Supplemental Digital Content, http://links.lww.com/MD/L940; Supplementary Figure 4, Supplemental Digital Content, http://links.lww.com/MD/L942). Even after the removal of outliers, the MR-PRESSO results did not support the presence of heterogeneous SNPs (Supplementary Table S4, Supplemental Digital Content, http://links.lww.com/MD/L934). After excluding metabolites with less significance (*P* < .05), our analysis revealed that both the Cochran-*Q* test and MR-Egger intercept test provided substantial evidence (*P* > .05) supporting the absence of heterogeneity and pleiotropy, as illustrated in Table 1. Furthermore, all our estimates demonstrated a remarkable level of statistical power, exceeding 0.8 (as indicated in Table 1). These findings prominently emphasize the robustness and reliability of our methodology, therefore further strengthening the validity of our conclusions. Collectively, these results firmly support the notion that our study offers valuable insights into the causal relationship between blood metabolites and PE. We further conducted LOO analysis, which revealed no high-influence SNPs capable of biasing the pooled effect estimates (Supplementary Figure 1, Supplemental Digital Content, http://links.lww.com/MD/L939; Supplementary Figure 3, Supplemental Digital Content, http://links.lww.com/MD/L941). These blood metabolites were considered as potential candidates for further investigation. The reverse MR analysis did not uncover any significant causal effects of PE on serum metabolites, as indicated in Supplementary Table S5, Supplemental Digital Content, http://links.lww.com/MD/L935. Furthermore, our comprehensive analysis not only confirmed the lack of significant heterogeneity or pleiotropy among the IVs but also indicated that there is no compelling evidence to support a reciprocal causal relationship between serum metabolites and the occurrence of preeclampsia or eclampsia.

**Table 1:** Supplementary and sensitivity analyses for causality from blood metabolites on PE.

Trait	Metabolites		MR analysis			Heterogeneity		Pleiotropy		Power
			Methods	OR (95% CI)	*P*	*Q*	*P*	Intercept	*P*	
Preeclamps	Amino acid									
		Isoleucine	ME	1.40 (0.12–16.71)	.79	12.59	.63	0.01	.45	1.00
			WM	2.42 (0.53–10.97)	.25					
		Citrulline	ME	0.23 (0.05–1.15)	.08	42.89	.52	0.01	.35	1.00
			WM	0.36 (0.15–0.87)	.02					
		Phenol sulfate	ME	1.36 (0.51–3.66)	.55	10.03	.82	0.01	.48	1.00
			WM	1.56 (0.93–2.60)	.09					
		Dimethylarginine	ME	0.21 (0.03–1.53)	.13	41.28	.13	0.01	.47	1.00
			WM	0.35 (0.12–1.01)	.05					
	Peptide									
		γ-glutamylglutamine	ME	0.67 (0.22–2.03)	.49	22.87	.24	−0.01	.50	1.00
			WM	0.45 (0.18–1.14)	.09					
		γ-glutamyltyrosine	ME	0.16 (0.01–2.19)	.18	64.31	.01	0.01	.48	1.00
			WM	0.44 (0.18–1.08)	.07					
		X-14304–leucylalanine	ME	0.37 (0.17–0.80)	.02	18.83	.40	0.03	.07	1.00
			WM	0.71 (0.49–1.02)	.06					
	Carbohydrate									
		Lactate	ME	0.12 (0.01–1.49)	.13	9.55	.48	0.01	.65	1.00
			WM	0.29 (0.08–1.03)	.05					
		Glucose	ME	0.30 (0.09–1.01)	.06	34.02	.56	0.01	.34	1.00
			WM	0.47 (0.17–1.30)	.15					
		1,5-anhydroglucitol	ME	6.01 (2.14–16.89)	.00	36.04	.17	−0.02	.02	1.00
			WM	2.41 (1.31–4.45)	.00					
	Nucleotide									
		Inosine	ME	0.87 (0.74–1.03)	.14	2.93	.97	0.00	.92	1.00
			WM	0.86 (0.73–1.01)	.07					
	Lipid									
		1-arachidonoyl-glycerophosphocholine	ME	0.54 (0.22–1.36)	.21	24.44	.18	0.00	.93	1.00
			WM	0.51 (0.28–0.91)	.02					
Eclamps	Amino acid									
		Phenylacetate	ME	1.04 (0.59–1.82)	.90	5.78	.67	−0.01	.79	1.00
			WM	0.96 (0.59–1.55)	.86					
		1-palmitoylglycerol	ME	1.65 (0.14–19.30)	.70	8.24	.69	−0.05	.47	1.00
			WM	2.09 (0.80–5.43)	.13					
		Indoleacetate	ME	0.75 (0.43–1.32)	.33	9.03	.97	−0.02	.46	1.00
			WM	0.81 (0.48–1.36)	.42					
		3-lactate	ME	0.66 (0.14–3.03)	.60	18.68	.61	−0.01	.90	1.00
			WM	1.22 (0.52–2.83)	.65					
		Tryptophan betaine	ME	0.93 (0.52–1.69)	.83	13.83	.24	0.07	.44	0.95
			WM	0.95 (0.74–1.23)	.72					
	Carbohydrate									
		Arabinose	ME	0.94 (0.54–1.63)	.83	0.56	.91	−0.04	.62	0.99
			WM	1.04 (0.70–1.53)	.85					
	Peptide									
		γ-glutamylglutamine	ME	0.67 (0.22–2.03)	.49	24.74	.17	0.00	.96	1.00
			WM	0.45 (0.18–1.14)	.09					
	Energy									
		Malate	ME	3.91 (0.30–50.95)	.31	12.18	.67	0.03	.72	1.00
			WM	1.35 (0.53–3.44)	.52					

### 3.2. Genetic correlation and direction validation

The results of the LDSC analysis provided limited evidence for a genetic correlation between preeclampsia and several blood metabolites, including isoleucine (Rg = NA, se = NA, *p* = NA), citrulline (Rg = 0.011, se = 0.087, *P* = .091), phenol sulfate (Rg = NA, se = NA, *p* = NA), dimethylarginine (Rg = −0.219, se = 0.183, *P* = .231), γ-glutamylglutamine (Rg = 0.297, se = 0.283, *P* = .293), X-14304–leucylalanine (Rg = NA, se = NA, *p* = NA), lactate (Rg = NA, se = NA, *p* = NA), glucose (Rg = 0.207, se = 0.145, *P* = .153), inosine (Rg = 0.006, se = 0.179, *P* = .971), and 1-arachidonoyl-glycerophosphocholine (Rg = −0.163, se = 0.120, *P* = .172). These findings suggest that any shared genetic components had minimal impact on the MR estimates (Supplementary Table S6, Supplemental Digital Content, http://links.lww.com/MD/L936). To further explore the potential causal relationships between these metabolites and preeclampsia, additional analyses and investigations are warranted. Based on LDSC analysis, we observed little genetic correlation between eclampsia and several blood metabolites, including phenylacetate (Rg = NA, se = NA, *p* = NA), 1-palmitoylglycerol (Rg = −0.742, se = 0.709, *P* = .296), indoleacetate (Rg = 1.793, se = 2.782, *P* = .519), 3-lactate (Rg = −1.434, se = 0.790, *P* = .069), tryptophan betaine (Rg = −0.307, se = 1.013, *P* = .762), arabinose (Rg = NA, se = NA, *p* = NA), γ-glutamylglutamine (Rg = −0.837, se = 1.974, *P* = .671), and malate (Rg = 0.596, se = 1.328, *P* = .654). These results suggest that the MR estimates are unlikely to be confounded by shared genetic components (Supplementary Table S6, Supplemental Digital Content, http://links.lww.com/MD/L936). These findings provide valuable insights into the genetic basis of these metabolic traits, which may have important implications for the prevention and treatment of eclampsia and related conditions.

### 3.3. Confounding analysis and MVMR

**Figure 3.**
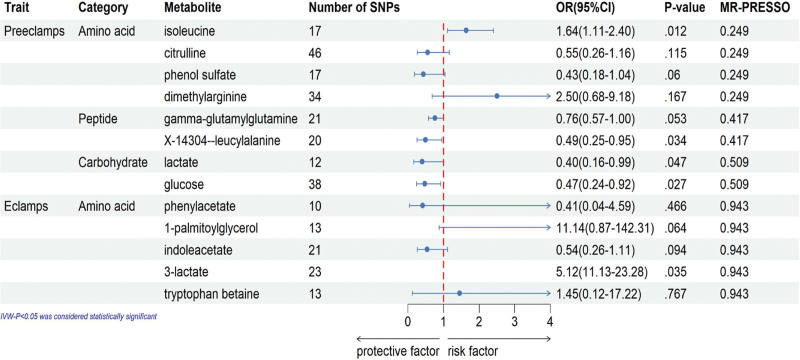
Multivariable MR analysis of the final identified blood metabolites. 95% CI = 95% confidence interval, IVW = inverse variance weighted, MR-PRESSO = MR-Pleiotropy RESidual Sum and Outlier, MVMR = Multivariable Mendelian randomization, OR = odds ratio.

Although confounding serum metabolites have been eliminated by sensitivity analysis, to meet the hypothesis that the IVs are independent of confounders, we needed to examine all SNPs of excluded metabolites to ensure that all SNPs were not associated with common risk factors for PE. Meanwhile, after excluding phenylacetate unrelated to risk factors, we found a total of 8 SNP associated with risk factors for eclampsia (Supplementary Table S7, Supplemental Digital Content, http://links.lww.com/MD/L937). To assess the specific influence of the exposure on the outcome while considering potential interrelationships among multiple exposures, we employed a sophisticated statistical technique known as MVMR analysis. This analysis involved applying a multiplicative inverse variance weighting approach with multivariate random effects, as illustrated in Figure 3. By utilizing this robust methodology, we were able to account for the complex relationships between various exposures and determine the direct impact of the exposure of interest on the outcome variable. The MVMR estimates obtained through the IVW method indicated that isoleucine, X-14304–leucylalanine, lactate, and glucose genetically predicted levels have independent direct effects on preeclampsia. Furthermore, 3-lactate was found to directly affect eclampsia, independent of other metabolites.

### 3.4. Metabolic pathway analysis

From the analysis of 10 established metabolites, we discovered 2 metabolic pathways that could potentially contribute to the development of preeclampsia. Additionally, 3 out of the 8 identified metabolites associated with eclampsia are likely involved in their metabolism (Supplementary Table S8, Supplemental Digital Content, http://links.lww.com/MD/L938). Importantly, lactate was found to be involved in the metabolic pathways associated with both PE. This finding strongly implies that lactate, in conjunction with its interconnected metabolic pathways, holds significant importance in the development and progression of both PE. This suggests that a comprehensive understanding of the involvement of lactate and its associated metabolic processes holds great potential for unraveling the underlying mechanisms of these conditions and ultimately guiding novel therapeutic strategies.

## 4. Discussion

In this study, we performed an unbiased MR analysis using the summary statistics of serum metabolites from the largest GWAS meta-analysis (http://metabolomics.helmholtz-muenchen.de/gwas/) and the summary statistics of PE from the FinnGen consortium R9 release data to causally assess 486 blood metabolites and the risk of PE. As far as we know, this study stands as the initial endeavor to integrate metabolic phenotyping with genome analysis in order to probe the causal associations between human serum metabolites and PE. In our preliminary investigation, we employed genetic variations as IVs to perform an IVW analysis on 486 metabolites. This analysis unveiled a link between 23 metabolites and preeclampsia, of which 10 were successfully identified. Moreover, we identified 18 metabolites associated with eclampsia, among which 8 were previously reported. These metabolites were then subjected to heterogeneity and sensitivity testing. Finally, we discovered that genetically determined high levels of citrulline, dimethylarginine, γ-glutamylglutamine, leucylalanine, lactate, glucose, inosine, and 1-arachidonoyl-glycerophosphocholine were associated with a lower risk of preeclampsia, whereas high levels of isoleucine and phenol sulfate were associated with an increased risk. Elevated concentrations of phenylacetate, 1-palmitoylglycerol, and indoleacetate have been shown to be associated with an escalated risk of developing eclampsia. On the other hand, higher levels of 3-lactate, tryptophan betaine, arabinose, γ-glutamylglutamine, and malate are correlated with a reduced risk of eclampsia. These observations suggest that identifying and elucidating the metabolic pathways involved in the production and regulation of these biomolecules may hold significant potential for improving our understanding of the pathogenesis of PE and identifying novel diagnostic and therapeutic targets.

PE is a pregnancy-related condition that impacts approximately 3%–5% of all pregnancies. It is characterized by both hypertension and endothelial dysfunction, resulting in widespread damage to various end-organs such as the liver, blood, kidneys, brain, and placenta. These pathological changes are thought to be triggered by a combination of genetic, environmental, and lifestyle factors, highlighting the complexity of this multifactorial disorder. Therefore, gaining a more comprehensive understanding of the underlying molecular mechanisms through further research is crucial for improving diagnostic accuracy and advancing treatment options. PE constitutes a significant global health burden as it represents a leading cause of maternal morbidity and mortality. The severe consequences associated with this condition include, but are not limited to, life-threatening complications such as liver rupture, renal failure, seizures, and stroke. The impact of preeclampsia extends beyond the affected individuals themselves, as it also poses a substantial risk to fetal well-being. Effective identification, timely intervention, and appropriate management strategies are paramount to mitigating the adverse outcomes associated with preeclampsia and improving maternal and neonatal health. Preeclampsia, the present mainstay of treatment for which remains limited, is a substantial contributor to adverse outcomes such as preterm birth, neonatal morbidity, and perinatal mortality.^[3,4]^ The occurrence of preeclampsia not only jeopardizes the health and well-being of the mother but also imposes a significant burden on the newborn, increasing the risk of various complications. Thus, enhancing our understanding of the pathophysiology underlying preeclampsia and developing more effective therapeutic approaches are crucial for reducing the associated adverse outcomes and improving both maternal and neonatal health. While prior research has indicated that associated serum metabolites play a role in the biological mechanism of PE, providing a potential opportunity for early disease treatment and prevention, their practical application for these purposes has been hampered by the lack of a clear cause-and-effect relationship between the 2. In order to provide guidance for disease screening and treatment, we undertook a pivotal MR study aimed at shedding light on the causal links between blood metabolites and preeclampsia and eclampsia, as well as the metabolic pathways involved.

During the early stages of pregnancy, successful trophoblast invasion and the subsequent modification of the uterine spiral artery play critical roles in facilitating optimal placental implantation and ensuring an adequate supply of nutrients and oxygen to support healthy fetal development. This intricate process involves the invasion of trophoblast cells into the maternal tissue, leading to the remodeling and enlargement of the spiral arteries. By adapting the uterine blood vessels to accommodate the increasing demands of the growing fetus, this intricate mechanism allows for the establishment of a functional placenta later in pregnancy, ensuring the delivery of essential nutrients and oxygen for the developing fetus. However, growing data suggest that insufficient energy supply leads to improper zygote implantation and remodeling of the superficial placental spiral arteries,^[30]^ implying that some metabolic anomalies may be the origin of PE, resulting in worldwide changes in the metabolome. Maternal metabolic changes can exert a significant influence on the progression and severity of these events, with the placenta acting as a mirror that reflects, to some degree, the maternal metabolic state. The dynamic interplay between maternal metabolic factors and placental function can impact various aspects of pregnancy, including nutrient transport, oxygenation, and hormone synthesis. Factors such as maternal obesity, gestational diabetes, and hypertension can alter the metabolic environment within the placenta, potentially compromising its ability to effectively support fetal growth and development. Understanding the intricate relationship between maternal metabolism and placental function is crucial for identifying potential therapeutic targets and interventions aimed at improving maternal and fetal health outcomes. As a result, PE is linked to different metabolic disorders like dyslipidemia, hyperuricemia, hyperglycemia, and insulin resistance.^[31]^ Carbohydrates are essential for maintaining normal cellular functions and act as critical substrates for mammalian energy metabolites. Glucose, a significant circulating carbohydrate, is primarily metabolized by placental trophoblasts to generate energy and can constitute 40%–60% of the glucose supply in the third trimester.^[32]^ Throughout the course of gestation, glucose assumes a pivotal role, serving not only as a fundamental energy source but also as a key regulator of early embryonic implantation and placental morphogenesis. This ubiquitous carbohydrate molecule is essential for providing the developing embryo with the necessary energy reserves for cellular proliferation and differentiation. Furthermore, glucose acts as a vital signaling molecule, orchestrating intricate molecular and cellular events critical for successful implantation and subsequent placental development. By elucidating the diverse roles and mechanisms by which glucose influences these early pregnancy processes, we open new avenues for understanding and potentially manipulating key factors involved in reproductive health and fertility. Specifically, during the first trimester of human pregnancy,^[33]^ research has shown that as much as 90% of glucose present is converted into lactate. This finding underscores the importance of glucose metabolism in supporting early fetal growth and development, and highlights the unique metabolic demands of pregnancy. Cystic embryo implantation demands elevated levels of lactate as the low pH nearby germ cells aids in the breakdown of the endometrium, preparing it for subsequent trophoblast invasion.^[34,35]^ Consequently, the production of lactate through the process of aerobic glycolysis becomes imperative for creating a favorable microenvironment in the uterus during early pregnancy. This lactate production not only aids in the preparation of the uterine lining for successful embryo implantation but also facilitates the invasion of trophoblast cells.^[36]^ It is through the efficient utilization of glucose metabolism via aerobic glycolysis that the necessary energy and metabolic substrates are provided, ensuring the proper development and progression of early pregnancy processes. This study demonstrates that the genetic susceptibility to high lactate levels reduces maternal risk of PE. Additionally, lactate contributes to 2 metabolic pathways that are significantly enriched, namely glycolysis/gluconeogenesis and pyruvate metabolism. Gene expression and lactate dehydrogenase (LDH) activity in the placenta affected by preeclampsia are significantly higher compared to a normal pregnancy.^[37]^ The human placenta has a propensity for lactate production and increased glucose consumption, with glycolysis playing a vital role as an energy pathway.^[38]^ LDH is an enzyme that plays a crucial role in catalyzing the conversion of pyruvate to lactate within the cytosol, thus contributing to the maintenance of cellular energy homeostasis. Simultaneously, a fraction of pyruvate is transported into the mitochondrial matrix via the mitochondrial pyruvate carrier, where it undergoes further metabolic processing. This coordinated interplay between LDH and mitochondrial pyruvate carrier ensures the efficient utilization of pyruvate as a substrate for energy production and metabolic pathways within the cell. By comprehending the intricate mechanisms of pyruvate metabolism and the interplay between these 2 essential components, we gain a deeper understanding of cellular bioenergetics and potential targets for therapeutic interventions in various metabolic disorders. In the matrix, pyruvate is metabolized by the pyruvate dehydrogenase complex, producing acetyl coenzyme A and various molecules that contribute to the regulation of cholesterol and lipid metabolism or participate in the citric acid cycle.^[39]^ Hypoxia triggers metabolic pathways, promoting glycolysis and augmenting LDH activity, which leads to the conversion of pyruvate into lactate.^[40]^ LDH is secreted as an intracellular enzyme, which is highly sensitive and useful for diagnosing various illnesses involving compromised cellular integrity. While glucose glycolysis serves as the key energy source for both the placenta and trophoblast, it is important to note that the metabolism of free fatty acids (FFA) also plays a crucial role in providing a significant energy supply for fetal growth and placental development. By efficiently utilizing FFAs, the developing fetus and placenta can meet their increasing energy demands and ensure proper development throughout gestation. Research suggests that the regulation of FFA metabolism is a complex process involving a range of metabolic pathways and signaling molecules, and further studies are needed to fully understand the intricate interplay between glucose and FFA metabolism during pregnancy. Nevertheless, the ability to harness and utilize both glucose and FFA metabolisms is essential for a successful pregnancy outcome. FFA also play an important role in metabolism and trophoblast function. Mallick et al (2021) carried out an in vitro study to compare the stimulating effects of several long-chain polyunsaturated fatty acids on placental angiogenesis and observed distinct potencies in the order as follows: docosahexaenoic acid > eicosapentaenoic acid > arachidonic acid > oleic acid.^[41]^ The researchers noted that different long-chain polyunsaturated fatty acids varied in their potency regarding angiogenesis promotion.

This study offers several advantages. First, it is the first MR investigation to establish a causal link between serum metabolites and PE, combining metabolomics and genomics. Second, the selected SNPs from reputable consortia have undergone peer review and possess sufficient sample sizes. Third, the used methods for assessing the causal relationship between metabolites and PE, such as IVW, WM, and MR-Egger, are robust. This analytical strategy is highly informative for future research. However, it is important to acknowledge the limitations of the current study. A potential constraint in our study is the relatively small number of SNPs examined on a genome-wide scale. To mitigate this limitation, we made a slight adjustment to the threshold used in the MR analysis, a method commonly employed in similar investigations. This approach has been taken in previous research to enhance statistical power and enable the inclusion of a broader range of SNPs of interest. While this adjustment may introduce some level of variability, it allows for a more comprehensive examination of the relationship between genetic factors and the outcome of interest. Overall, our decision to relax the threshold in the MR analysis improves the robustness and scope of our findings. Another limitation is the small sample size, and the lack of a replication cohort analysis to validate our findings. Hence, it is imperative to increase the sample size in order to validate the robustness and reliability of our findings. Moreover, although the MR analysis offers valuable insights into the causal relationship, it is vital to emphasize that our results warrant further validation through rigorous Randomized Controlled Trials and fundamental studies before any practical application can be recommended. By conducting larger-scale studies and employing diverse research methodologies, we can establish a more comprehensive understanding of the underlying mechanisms and implications of our findings, which will ultimately enhance the translation of our research into practical interventions and clinical practice.

In conclusion, our MR analysis revealed a causal link between various blood metabolites and PE. This preliminary evidence suggests that these metabolites have an impact on the progression of PE and can be used to develop personalized explanations or markers for disease status. Moreover, these molecules present themselves as viable targets for future investigations, exhibiting great potential in advancing our understanding and exploration of their biological functions, mechanisms, and potential therapeutic applications. The identification of these molecules within our study not only expands our knowledge but also unlocks fresh avenues for further research. By delving deeper into the intricacies associated with these candidate molecules, we can unravel their intricate roles across a range of physiological processes and diseases. This endeavor will undoubtedly pave the way for significant breakthroughs and advancements within the realm of biomedical research. With their promising attributes, these molecules are poised to exert a profound influence, providing valuable insights into the development of innovative diagnostic tools, therapeutic strategies, and targeted interventions. However, the details of preventing and monitoring the progression of PE are still unclear. Therefore, further research on correlating metabolites is needed to obtain accurate indicators and strategies for preventing and treating the condition in the future.

## Acknowledgments

The authors express their gratitude to the UK-Biobank and the FinnGen consortiums for generously sharing their publicly available GWAS summary-level statistics. The authors thank all participants for their valuable contribution to this study.

## Author contributions

**Conceptualization:** Jiping Wei, Liyuan Huang, Yongfu Song, Xiaodan Lu.

**Data curation:** Jiping Wei, Liyuan Huang.

**Funding acquisition:** Xiaodan Lu.

**Investigation:** Jiping Wei, Liyuan Huang.

**Methodology:** Jiping Wei, Liyuan Huang, Yongfu Song, Xiaodan Lu.

**Software:** Jiping Wei, Liyuan Huang, Yongfu Song, Yongji Wang.

**Supervision:** Mingda Wu, Yan Guo, Xiaodan Lu.

**Validation:** Jiping Wei, Yongfu Song, Yongji Wang.

**Writing—original draft:** Jiping Wei.

**Writing—review & editing:** Jiping Wei, Liyuan Huang, Mingda Wu.

## Supplementary Material
























