# Supplementary material for: The relationship between human blood metabolites and preeclampsia-eclampsia: A Mendelian randomization study
Source: Medicine (Baltimore). 2024 Mar 29;103(13):e37505. doi: 10.1097/MD.0000000000037505 (PMC10977518; doi:10.1097/MD.0000000000037505)
Supplement: Supplementary file 4 [file medi-103-e37505-s004.pdf]

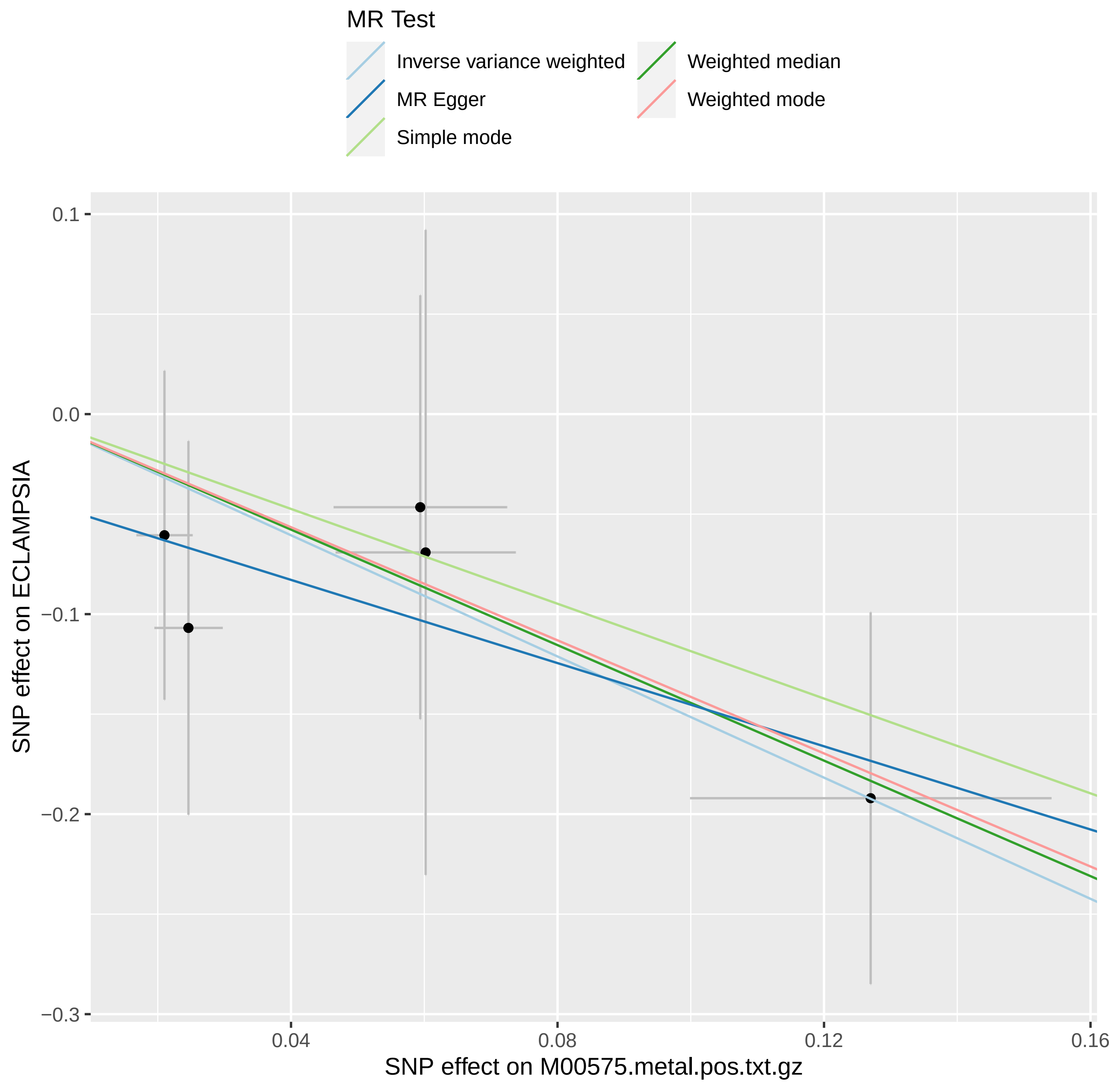

MR Test
Inverse variance weighted
MR Egger
Simple mode
Weighted median
Weighted mode
0.1
0.0
−0.1
−0.2
−0.3
0.04
0.08
0.12
0.16
SNP effect on ECLAMPSIA
SNP effect on M00575.metal.pos.txt.gz

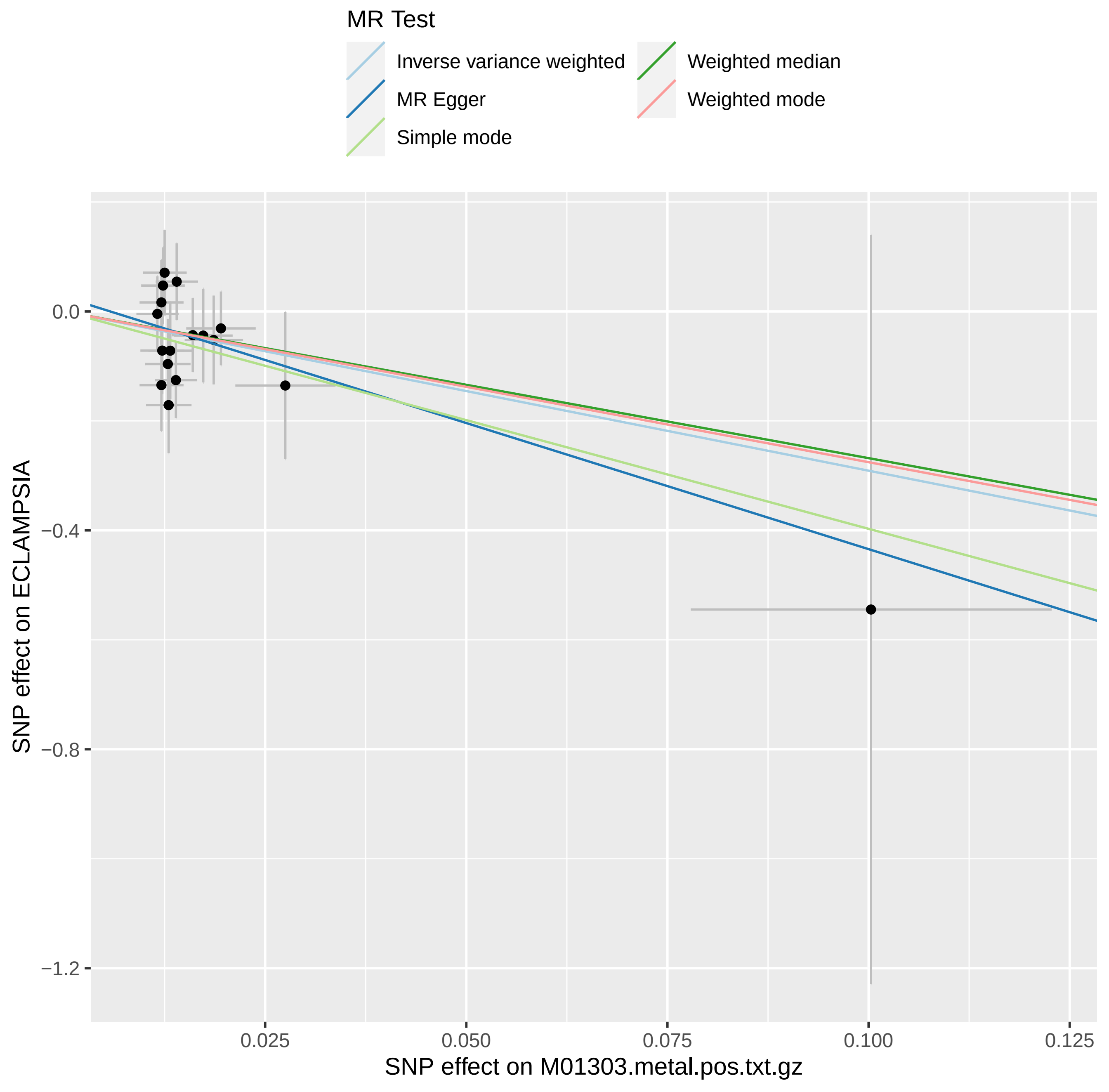
MR Test
Inverse variance weighted
MR Egger
Simple mode
Weighted median
Weighted mode
0.0
−0.4
−0.8
−1.2
SNP effect on ECLAMPSIA
0.025
0.050
0.075
0.100
0.125
SNP effect on M01303.metal.pos.txt.gz

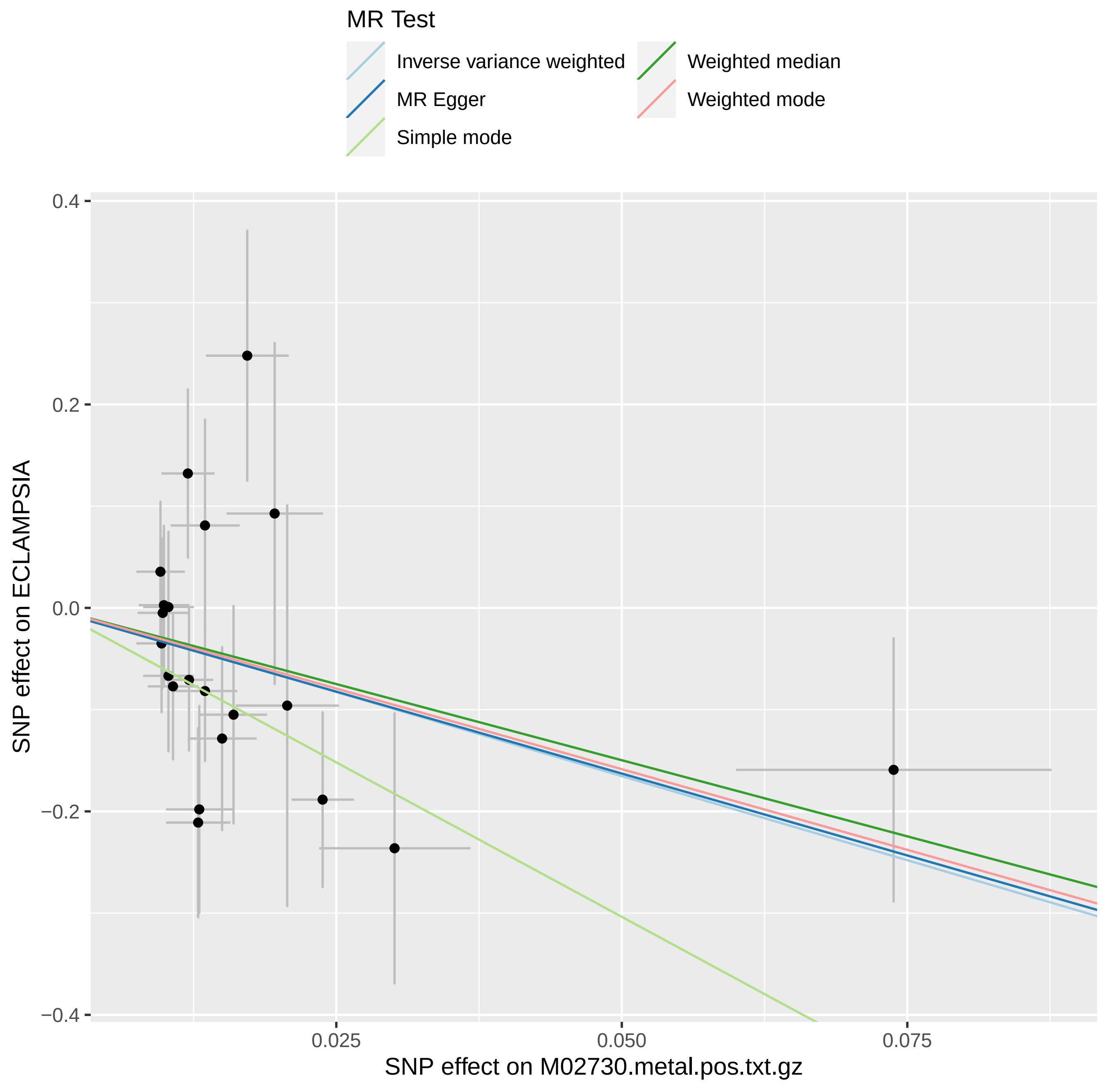
MR Test
Inverse variance weighted
MR Egger
Simple mode
Weighted median
Weighted mode
0.4
0.2
0.0
−0.2
−0.4
SNP effect on ECLAMPSIA
0.025
0.050
0.075
SNP effect on M02730.metal.pos.txt.gz

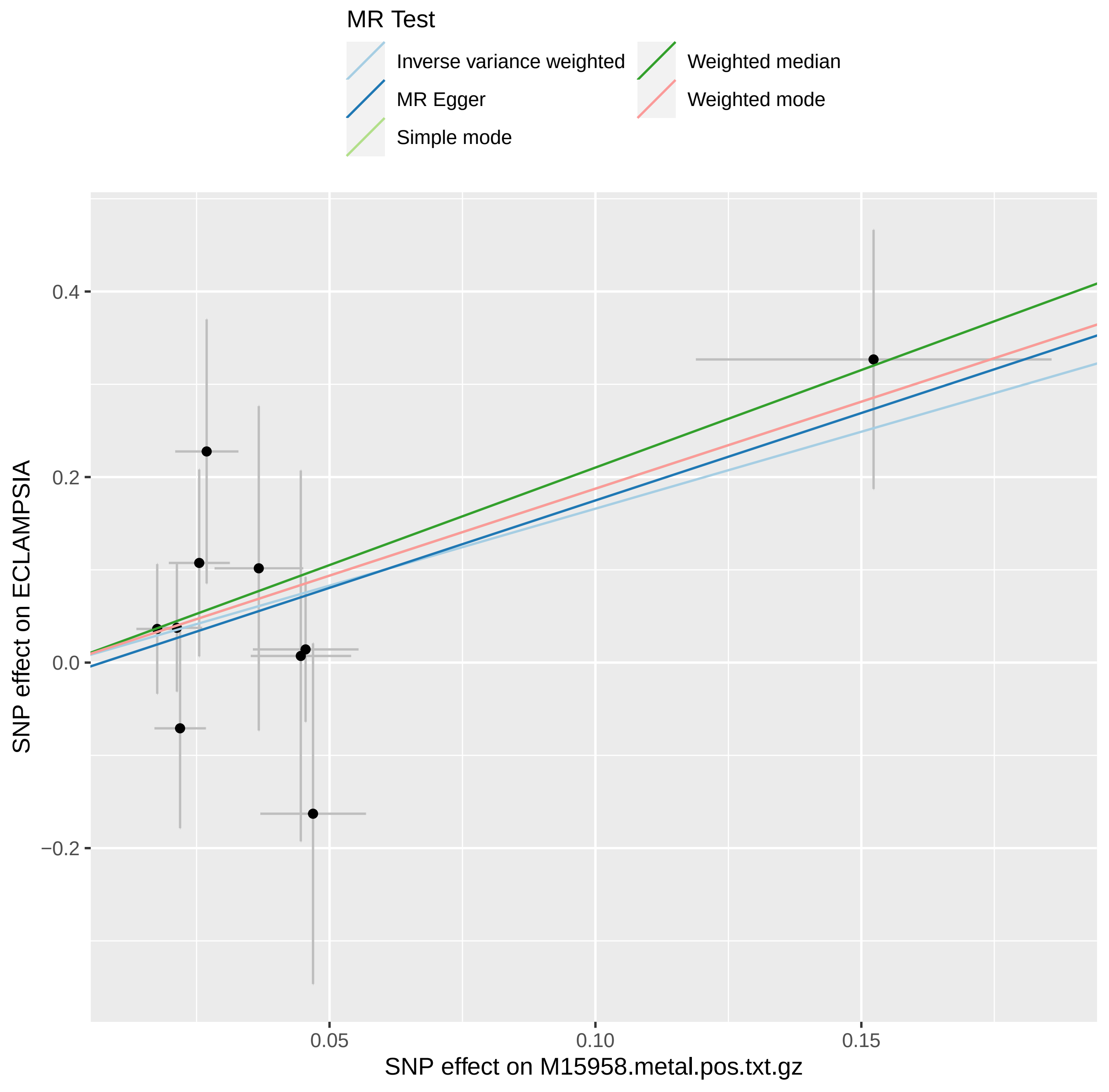

MR Test
Inverse variance weighted
MR Egger
Simple mode
Weighted median
Weighted mode
0.4
0.2
0.0
−0.2
0.05
0.10
0.15
SNP effect on ECLAMPSIA
SNP effect on M15958.metal.pos.txt.gz

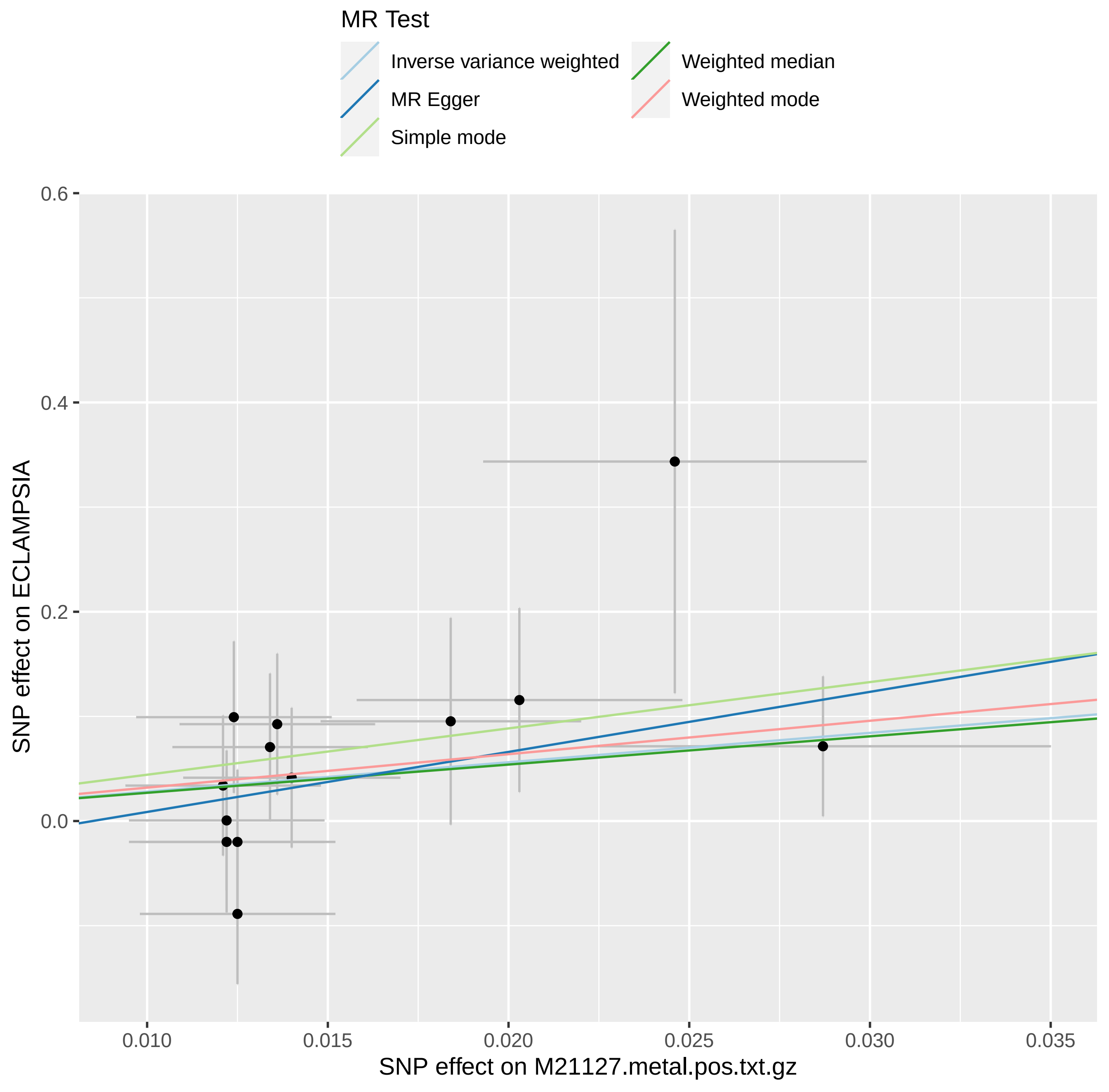
MR Test
Inverse variance weighted
MR Egger
Simple mode
Weighted median
Weighted mode
0.6
0.4
0.2
0.0
SNP effect on ECLAMPSIA
0.010
0.015
0.020
0.025
0.030
0.035
SNP effect on M21127.metal.pos.txt.gz

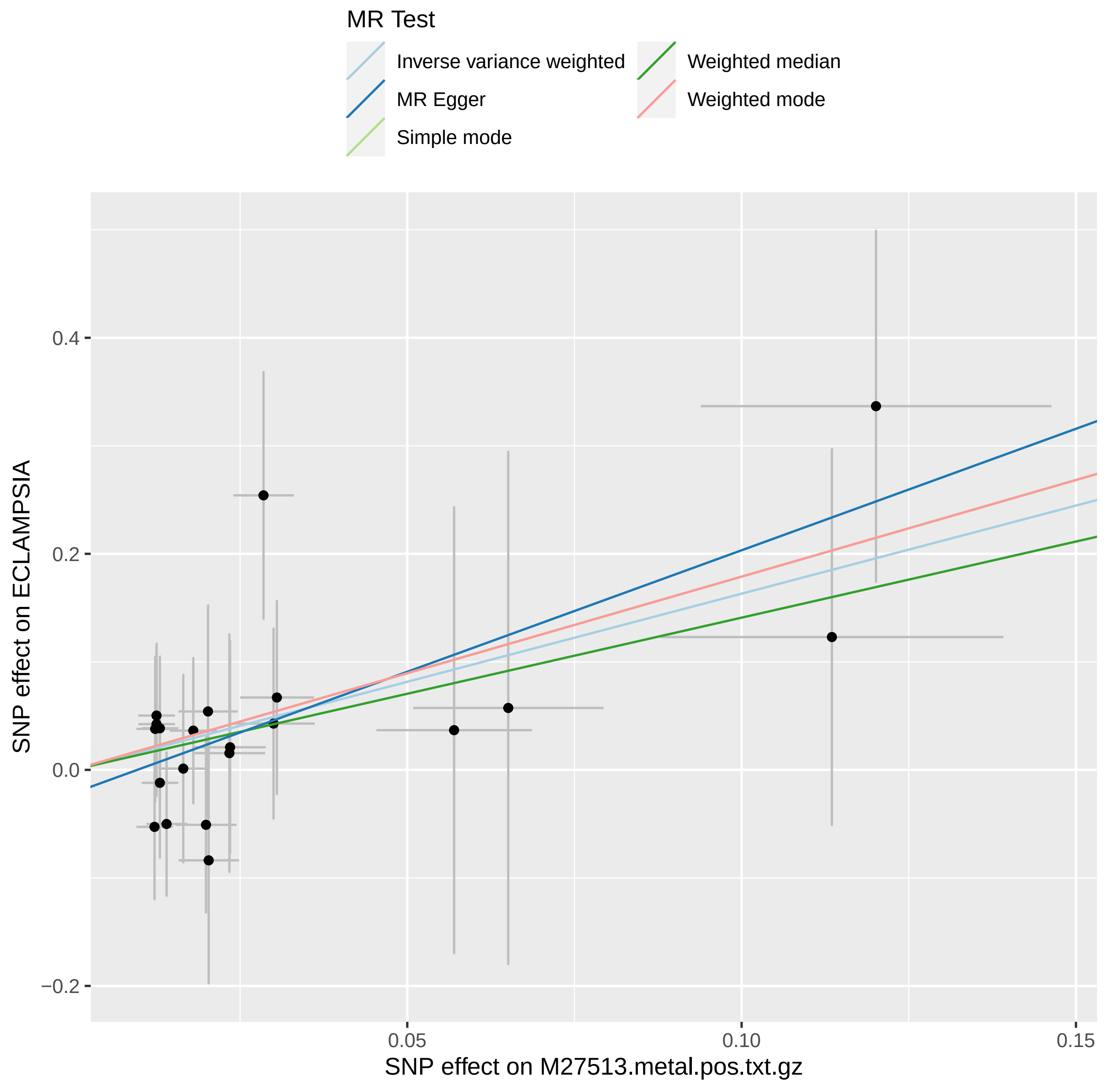

MR Test
Inverse variance weighted
MR Egger
Simple mode
Weighted median
Weighted mode
0.4
0.2
0.0
−0.2
0.05
0.10
0.15
SNP effect on ECLAMPSIA
SNP effect on M27513.metal.pos.txt.gz

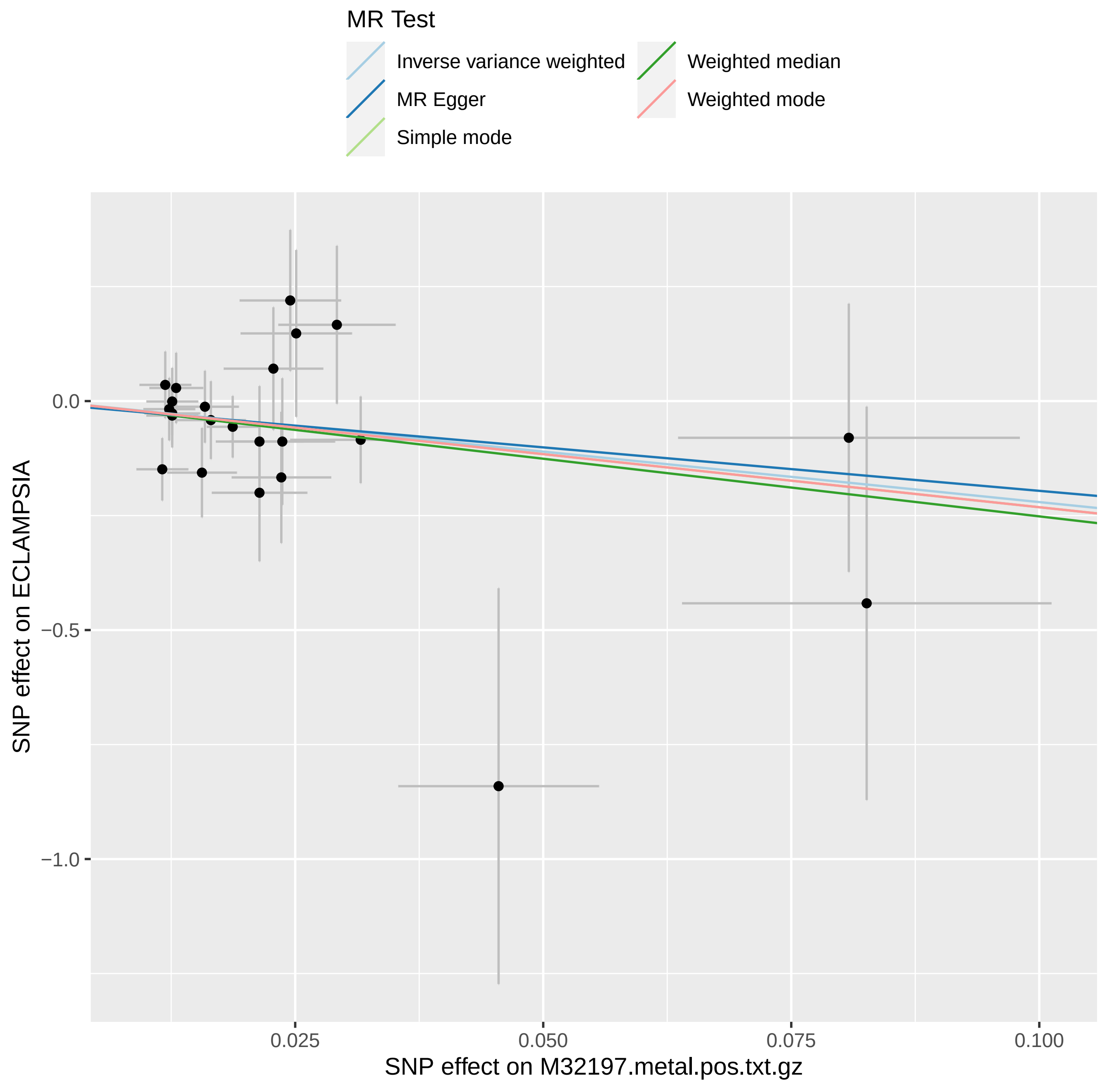
MR Test
Inverse variance weighted
MR Egger
Simple mode
Weighted median
Weighted mode
0.0
−0.5
−1.0
0.025
0.050
0.075
0.100
SNP effect on ECLAMPSIA
SNP effect on M32197.metal.pos.txt.gz

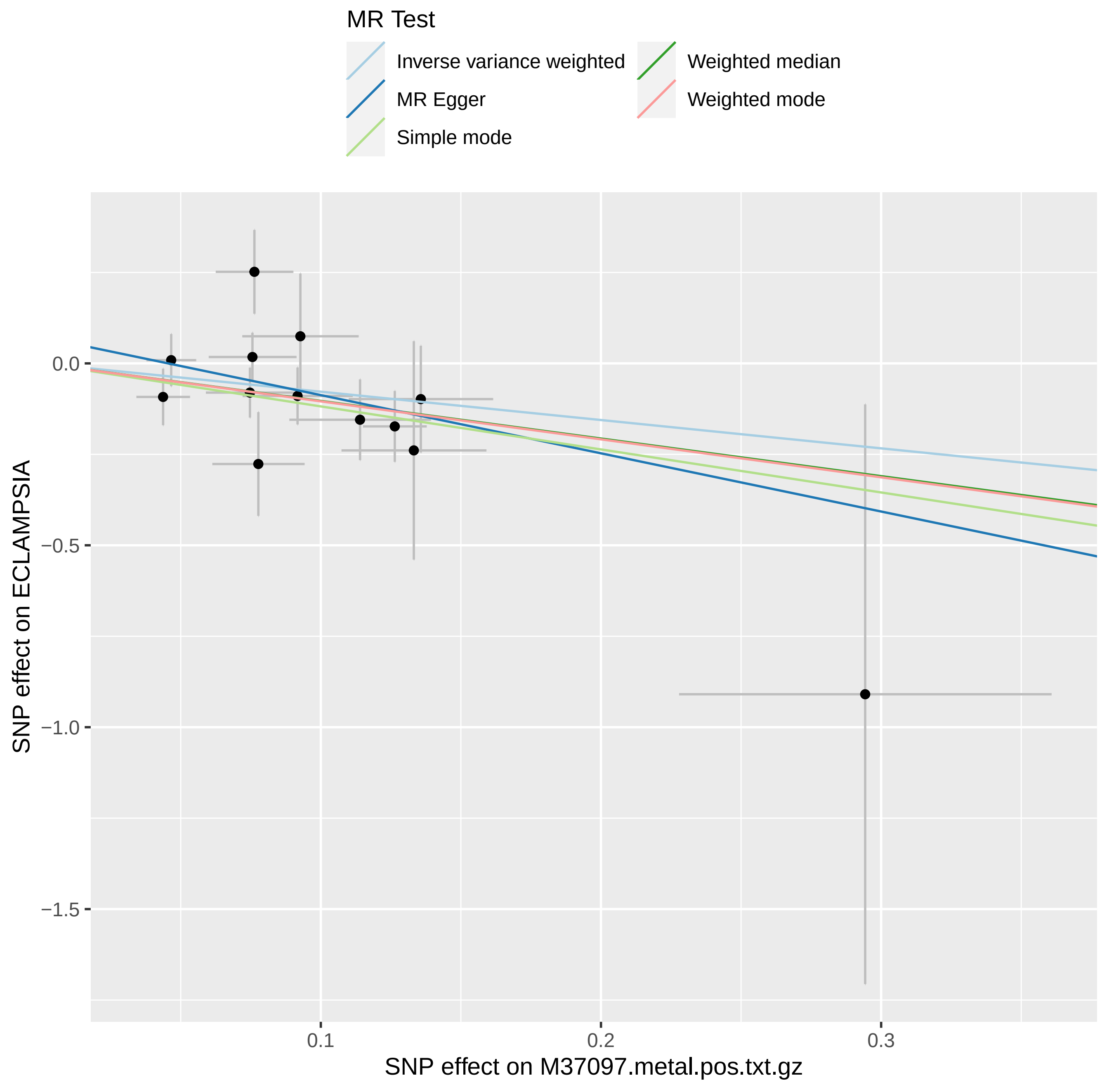
MR Test
Inverse variance weighted
MR Egger
Simple mode
Weighted median
Weighted mode
0.0
−0.5
−1.0
−1.5
0.1
0.2
0.3
SNP effect on ECLAMPSIA
SNP effect on M37097.metal.pos.txt.gz
