# Supplementary material for: The relationship between human blood metabolites and preeclampsia-eclampsia: A Mendelian randomization study
Source: Medicine (Baltimore). 2024 Mar 29;103(13):e37505. doi: 10.1097/MD.0000000000037505 (PMC10977518; doi:10.1097/MD.0000000000037505)
Supplement: Supplementary file 5 [file medi-103-e37505-s005.pdf]

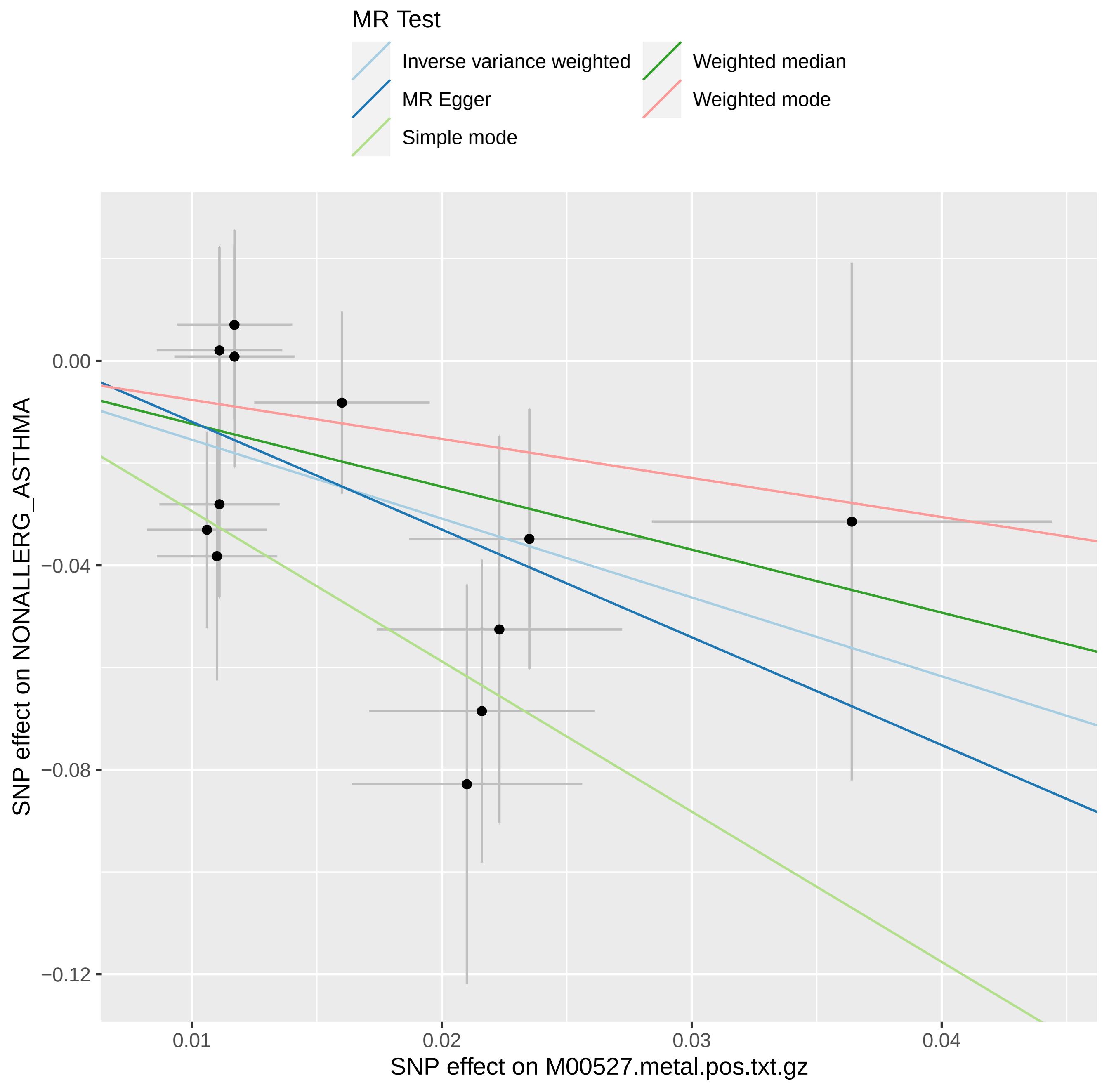
MR Test
Inverse variance weighted
MR Egger
Simple mode
Weighted median
Weighted mode
SNP effect on NONALLERG_ASTHMA
0.00
−0.04
−0.08
−0.12
0.01
0.02
0.03
0.04
SNP effect on M00527.metal.pos.txt.gz

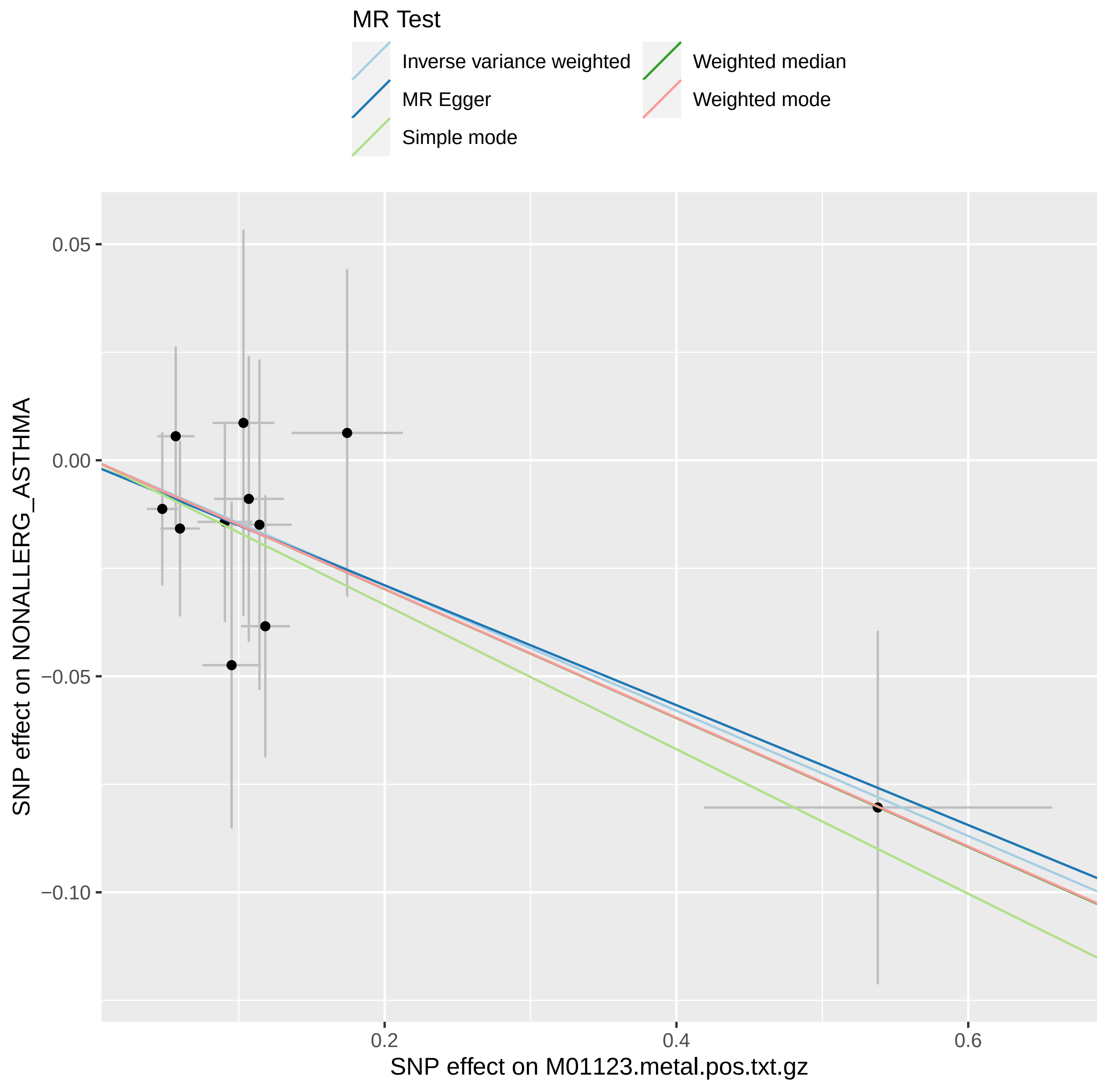

MR Test
Inverse variance weighted
MR Egger
Simple mode
Weighted median
Weighted mode
0.05
0.00
−0.05
−0.10
0.2
0.4
0.6
SNP effect on NONALLERG_ASTHMA
SNP effect on M01123.metal.pos.txt.gz

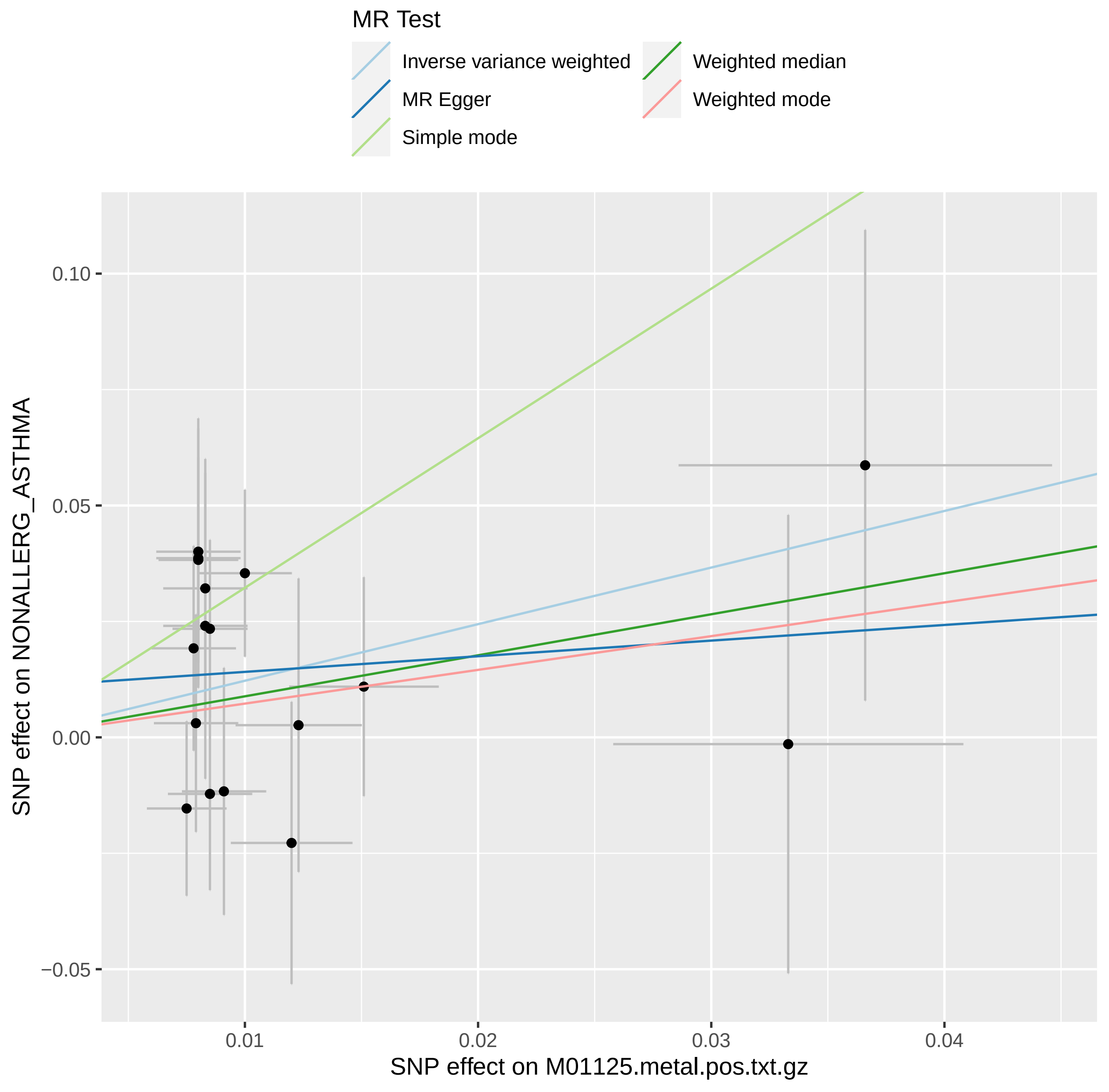

MR Test
Inverse variance weighted
MR Egger
Simple mode
Weighted median
Weighted mode
0.10
0.05
0.00
−0.05
0.01
0.02
0.03
0.04
SNP effect on NONALLERG_ASTHMA
SNP effect on M01125.metal.pos.txt.gz

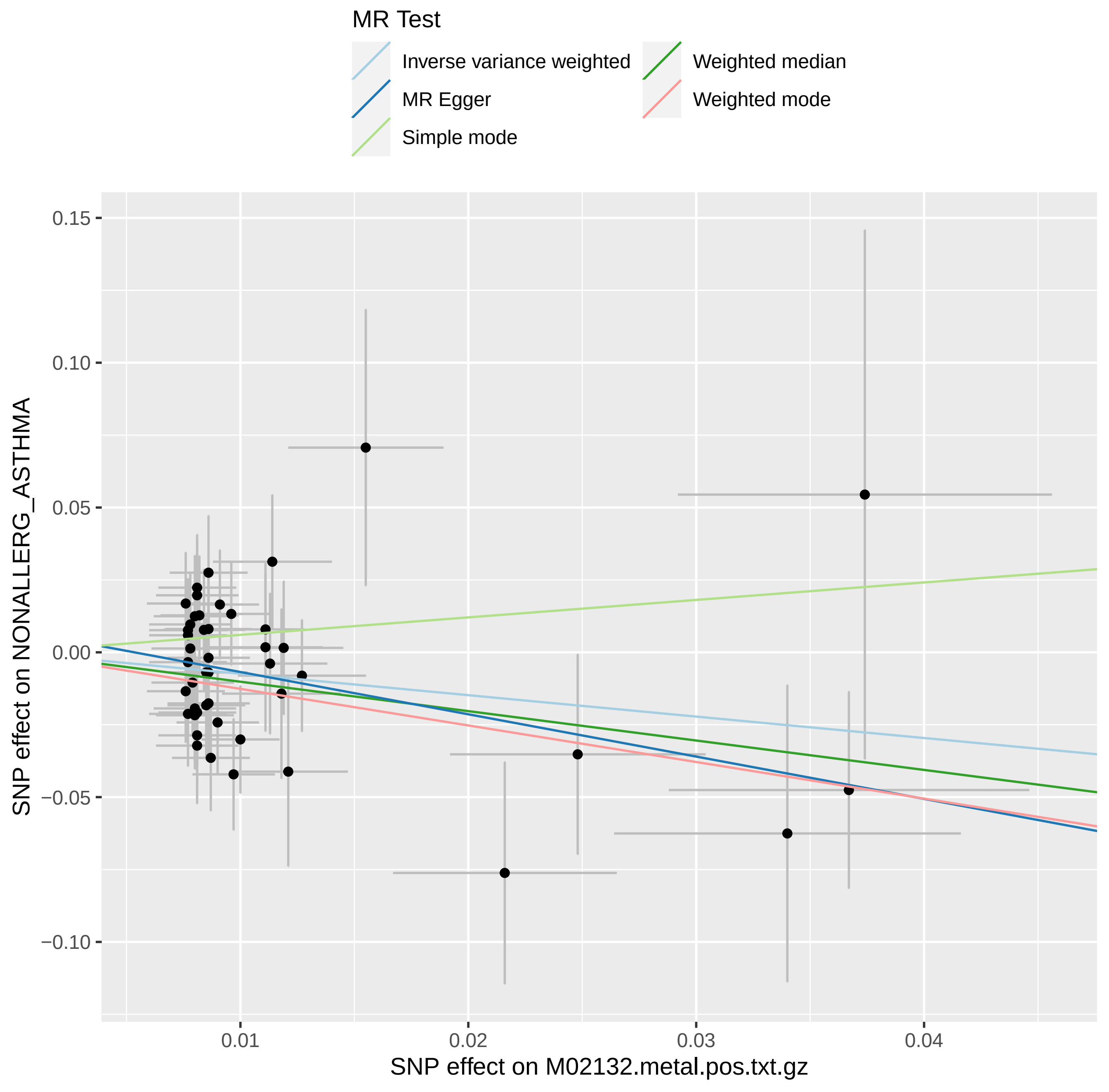
MR Test
Inverse variance weighted
MR Egger
Simple mode
Weighted median
Weighted mode
0.15
0.10
0.05
0.00
−0.05
−0.10
SNP effect on NONALLERG_ASTHMA
0.01
0.02
0.03
0.04
SNP effect on M02132.metal.pos.txt.gz

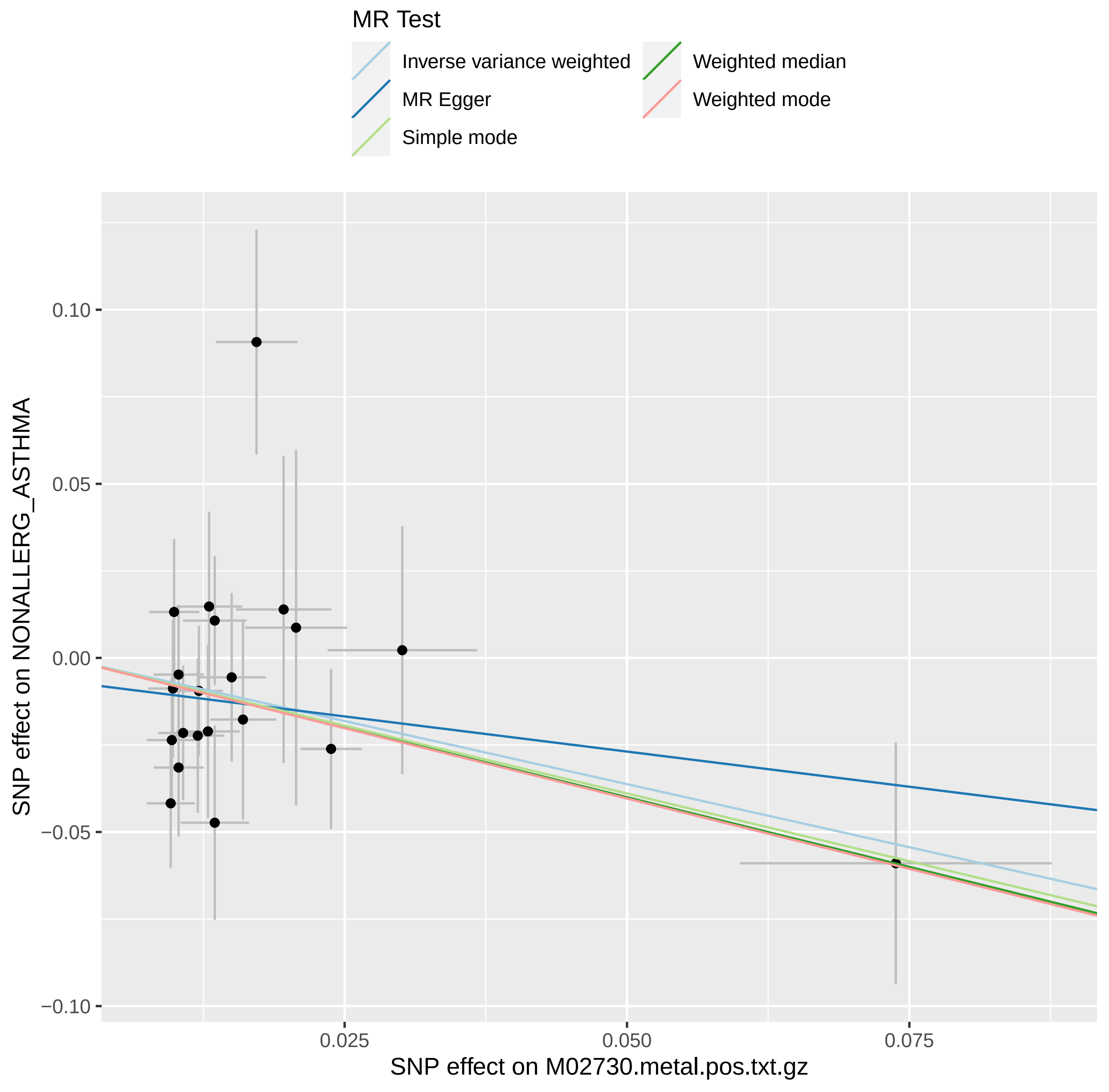

MR Test
Inverse variance weighted
MR Egger
Simple mode
Weighted median
Weighted mode
0.10
0.05
0.00
−0.05
−0.10
SNP effect on NONALLERG_ASTHMA
0.025
0.050
0.075
SNP effect on M02730.metal.pos.txt.gz

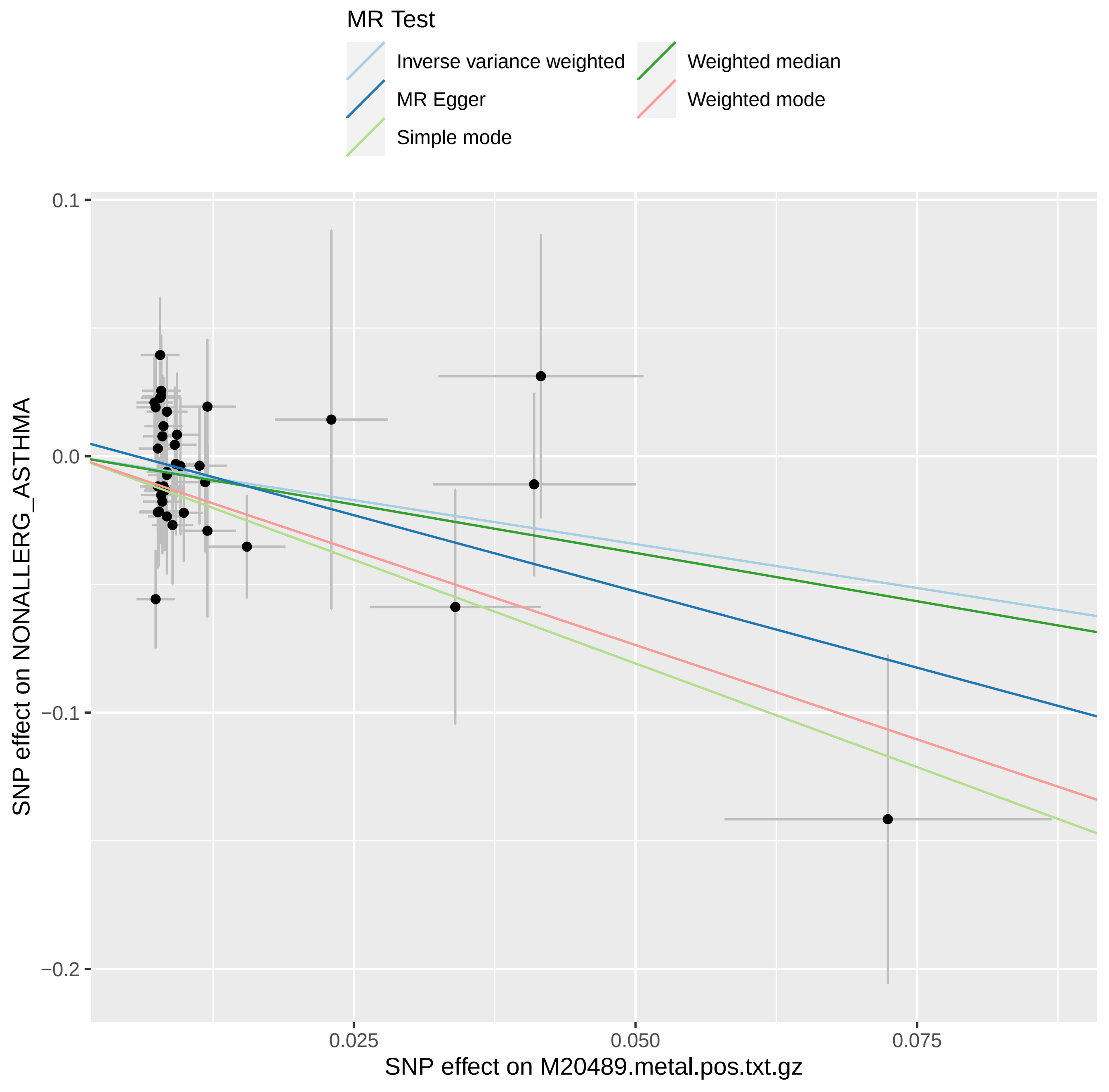
MR Test
Inverse variance weighted
MR Egger
Simple mode
Weighted median
Weighted mode
0.1
0.0
−0.1
−0.2
0.025
0.050
0.075
SNP effect on NONALLERG_ASTHMA
SNP effect on M20489.metal.pos.txt.gz

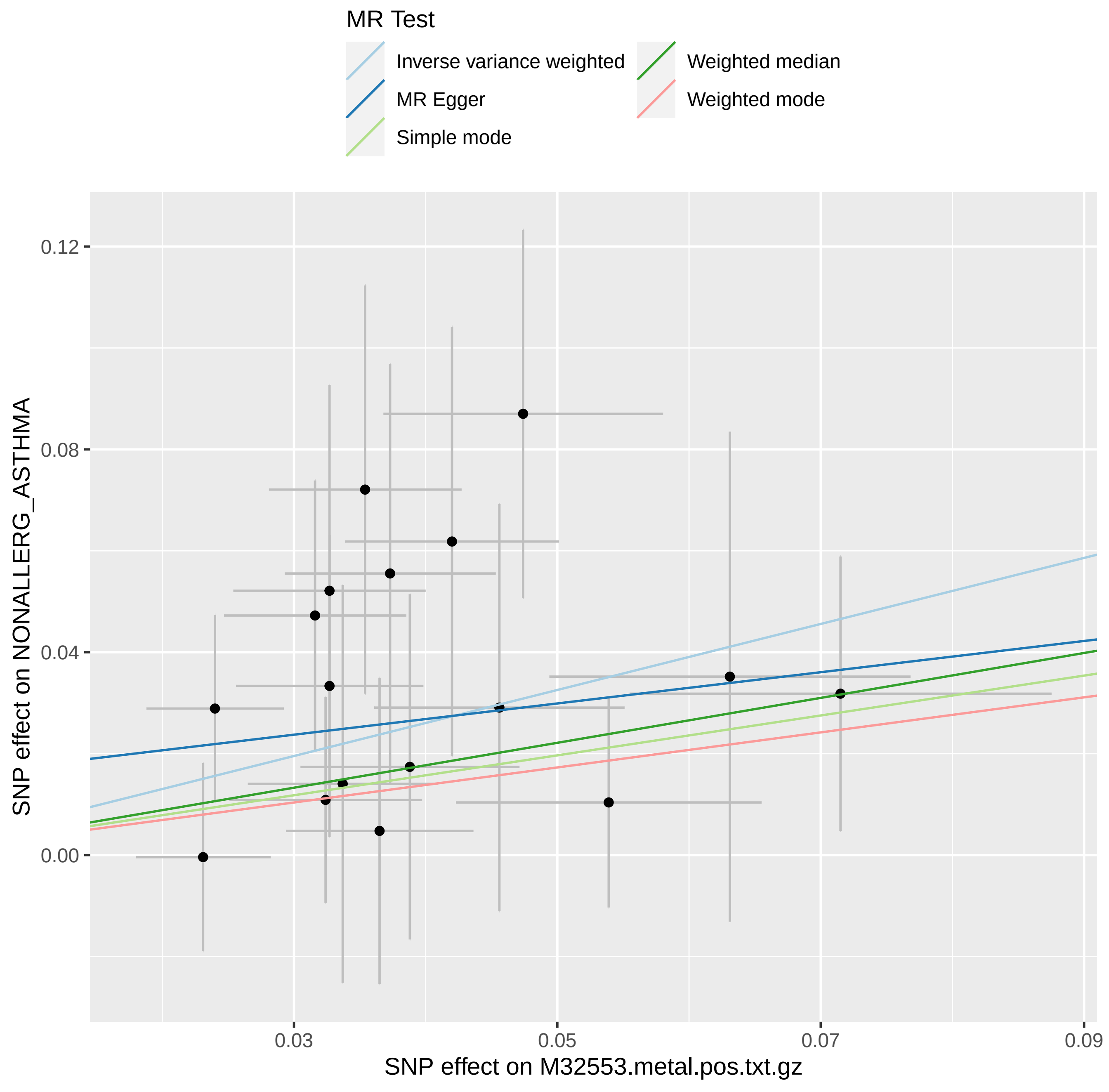

MR Test
Inverse variance weighted
MR Egger
Simple mode
Weighted median
Weighted mode
SNP effect on NONALLERG_ASTHMA
0.12
0.08
0.04
0.00
0.03
0.05
0.07
0.09
SNP effect on M32553.metal.pos.txt.gz

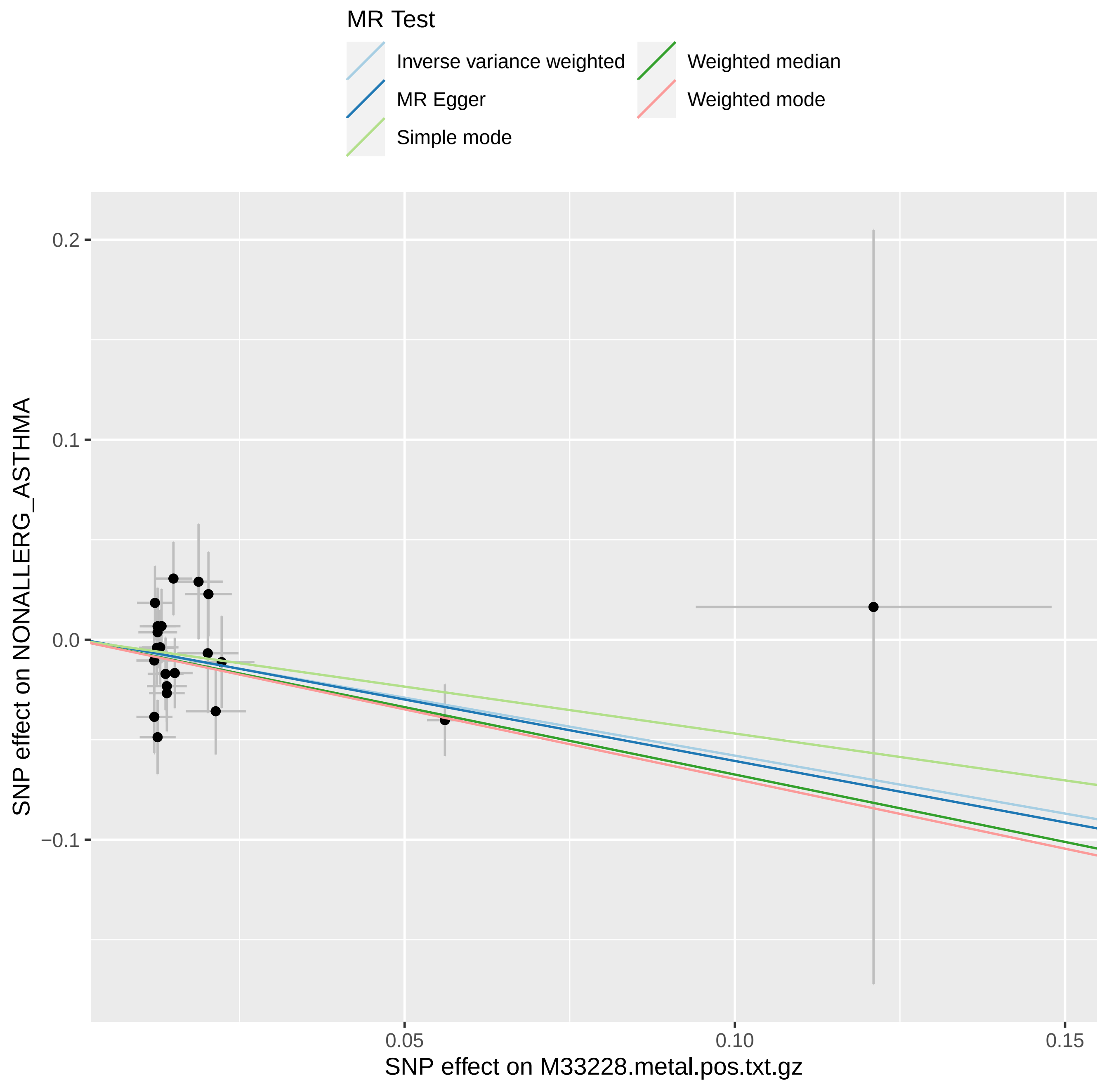

MR Test
Inverse variance weighted
MR Egger
Simple mode
Weighted median
Weighted mode
0.2
0.1
0.0
−0.1
0.05
0.10
0.15
SNP effect on NONALLERG_ASTHMA
SNP effect on M33228.metal.pos.txt.gz

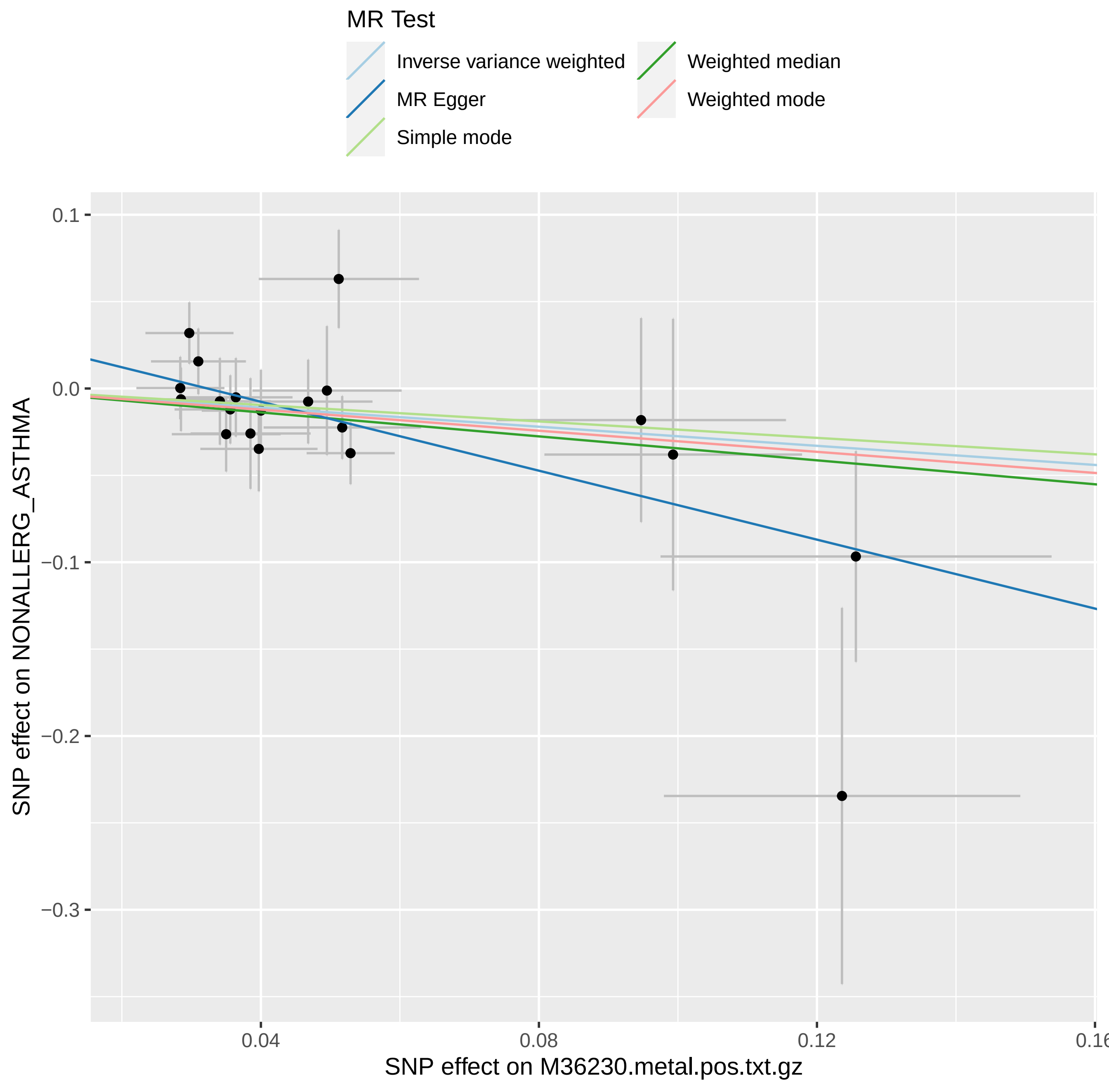

MR Test
Inverse variance weighted
MR Egger
Simple mode
Weighted median
Weighted mode
0.1
0.0
−0.1
−0.2
−0.3
0.04
0.08
0.12
0.16
SNP effect on NONALLERG_ASTHMA
SNP effect on M36230.metal.pos.txt.gz

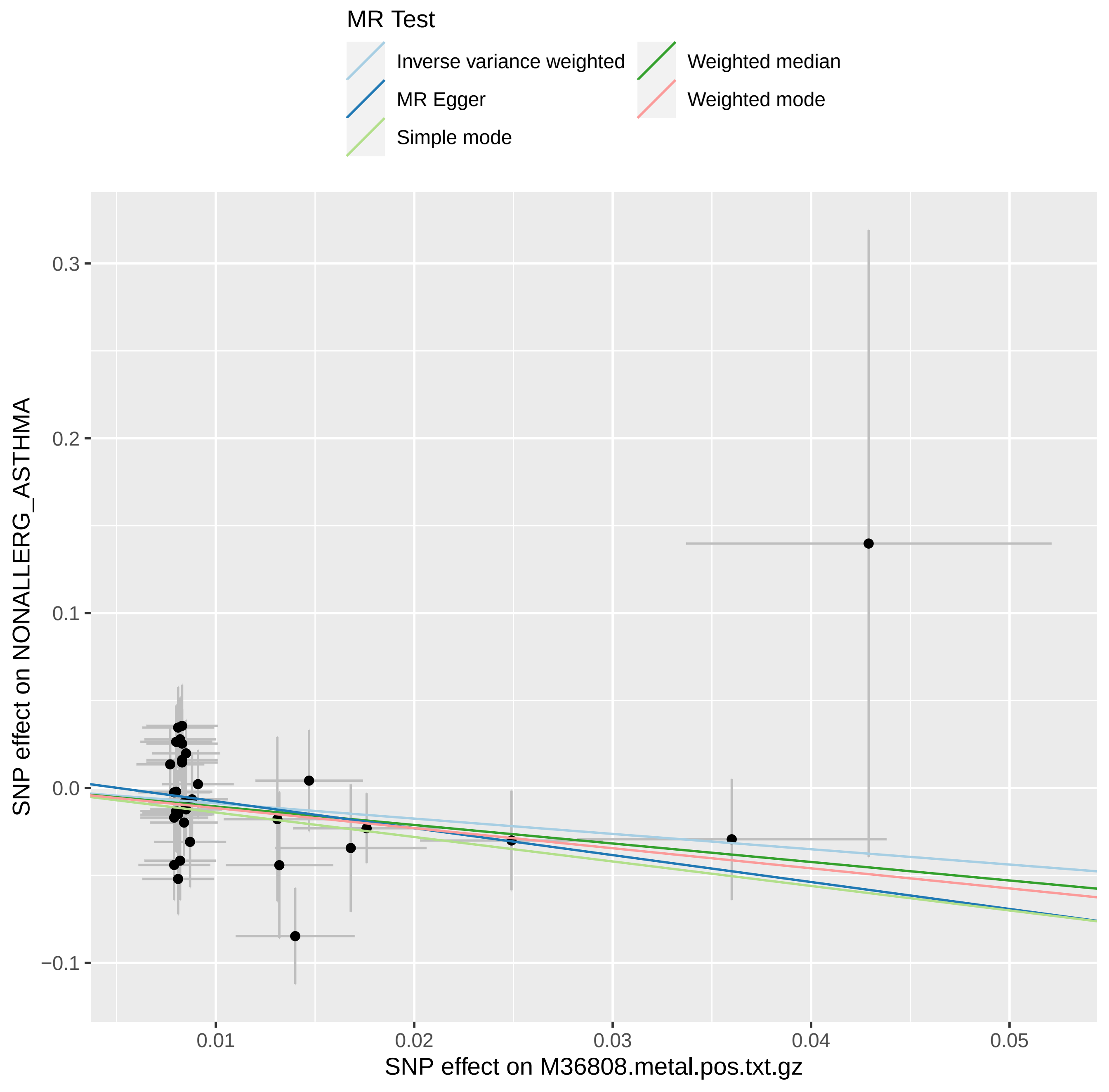
MR Test
Inverse variance weighted
MR Egger
Simple mode
Weighted median
Weighted mode
SNP effect on NONALLERG_ASTHMA
0.3
0.2
0.1
0.0
−0.1
0.01
0.02
0.03
0.04
0.05
SNP effect on M36808.metal.pos.txt.gz
