# Supplementary material for: The relationship between human blood metabolites and preeclampsia-eclampsia: A Mendelian randomization study
Source: Medicine (Baltimore). 2024 Mar 29;103(13):e37505. doi: 10.1097/MD.0000000000037505 (PMC10977518; doi:10.1097/MD.0000000000037505)
Supplement: Supplementary file 7 [file medi-103-e37505-s007.pdf]

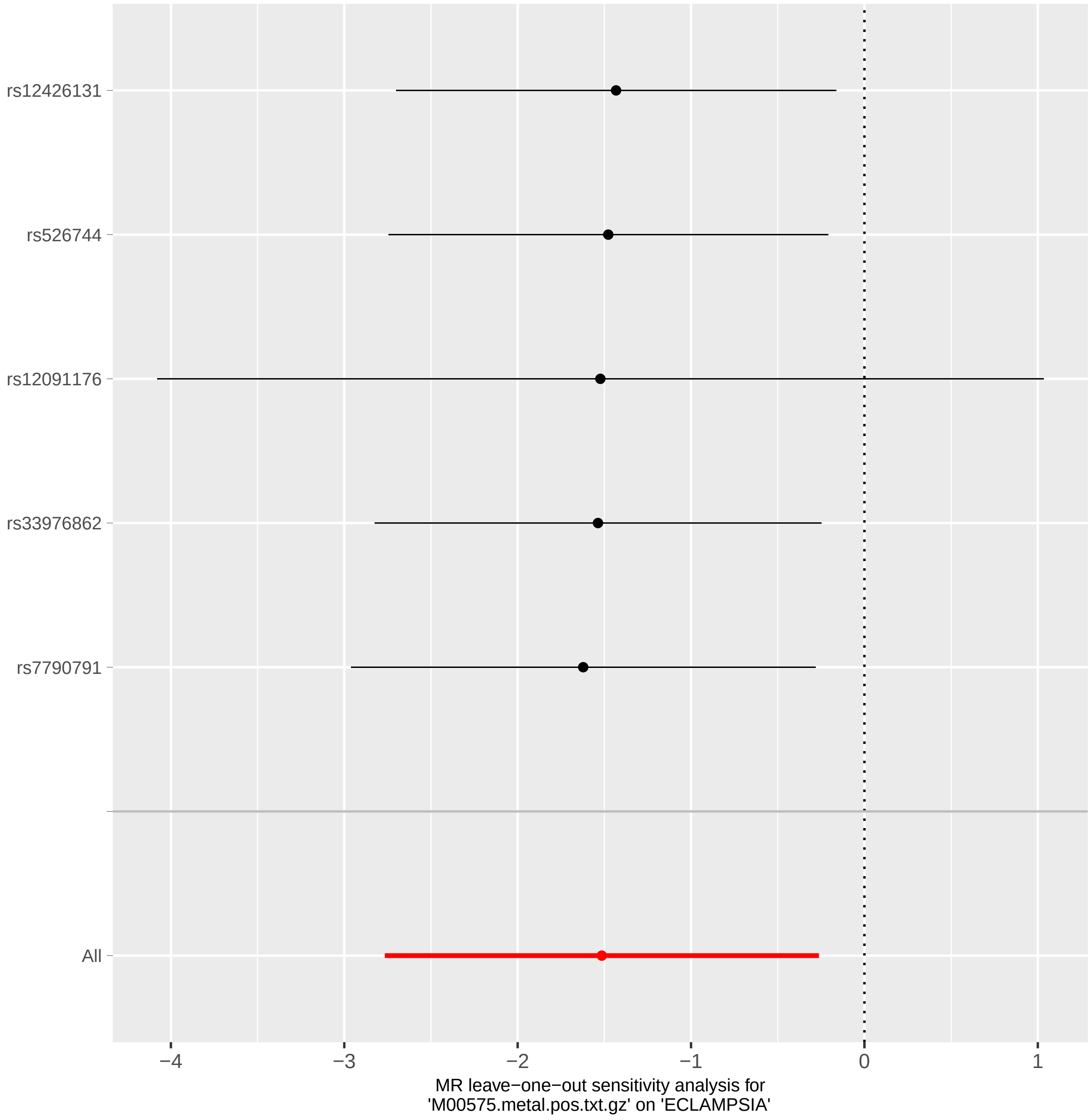

rs12426131
rs526744
rs12091176
rs33976862
rs7790791
All
−4
−3
−2
−1
0
1
MR leave−one−out sensitivity analysis for
'M00575.metal.pos.txt.gz' on 'ECLAMPSIA'

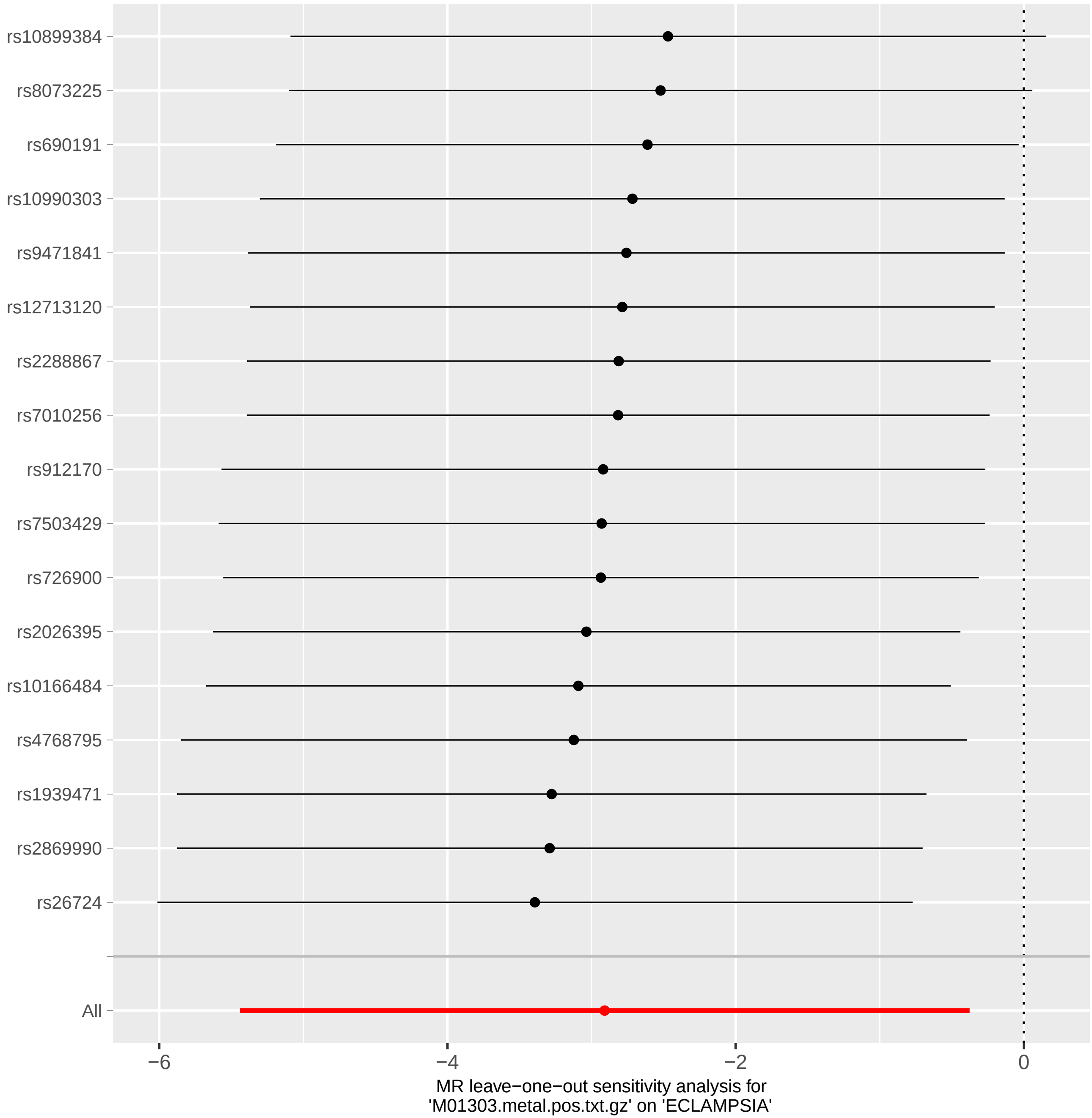
rs10899384
rs8073225
rs690191
rs10990303
rs9471841
rs12713120
rs2288867
rs7010256
rs912170
rs7503429
rs726900
rs2026395
rs10166484
rs4768795
rs1939471
rs2869990
rs26724
All
−6
−4
−2
0
MR leave−one−out sensitivity analysis for
'M01303.metal.pos.txt.gz' on 'ECLAMPSIA'

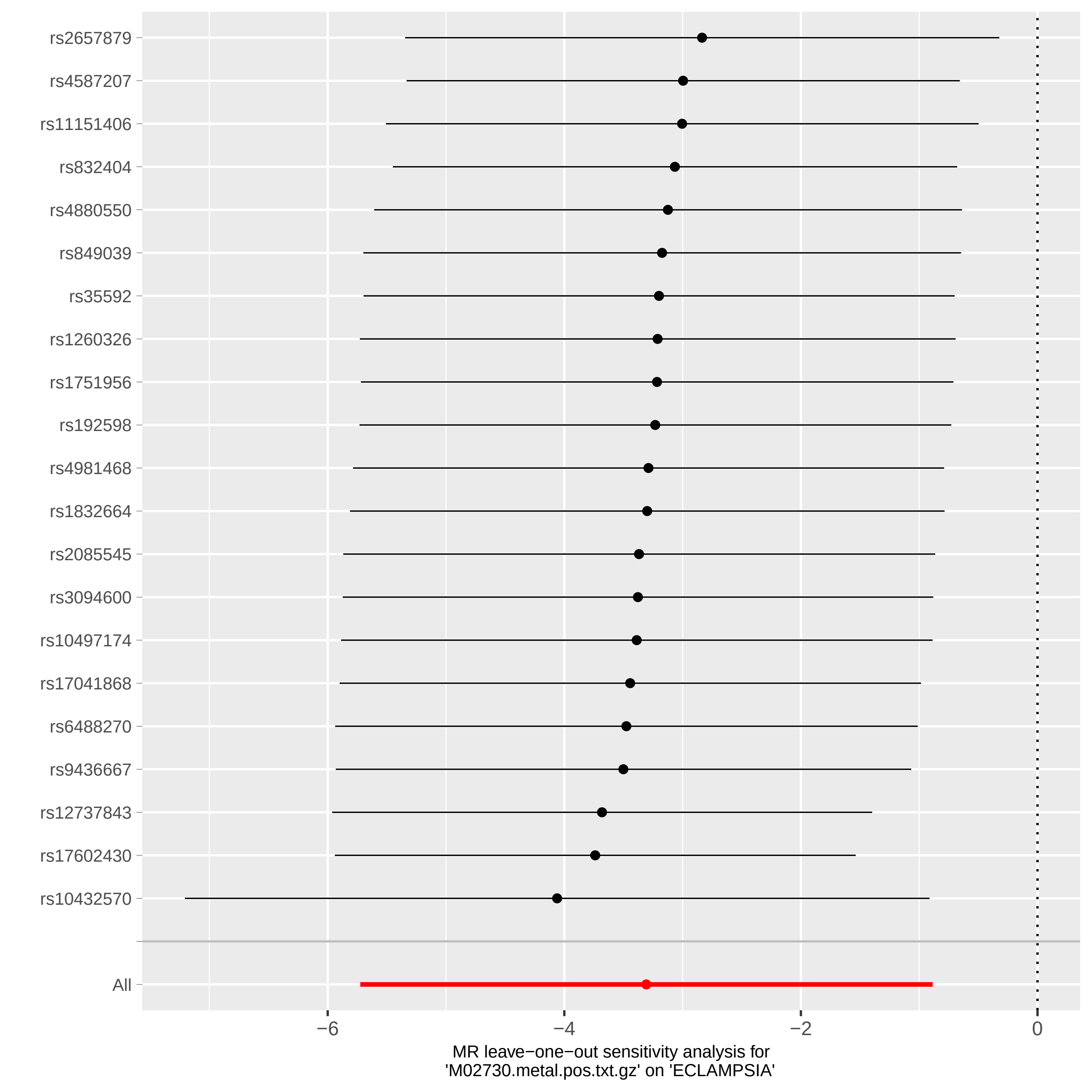
rs2657879
rs4587207
rs11151406
rs832404
rs4880550
rs849039
rs35592
rs1260326
rs1751956
rs192598
rs4981468
rs1832664
rs2085545
rs3094600
rs10497174
rs17041868
rs6488270
rs9436667
rs12737843
rs17602430
rs10432570
All
−6
−4
−2
0
MR leave−one−out sensitivity analysis for
'M02730.metal.pos.txt.gz' on 'ECLAMPSIA'

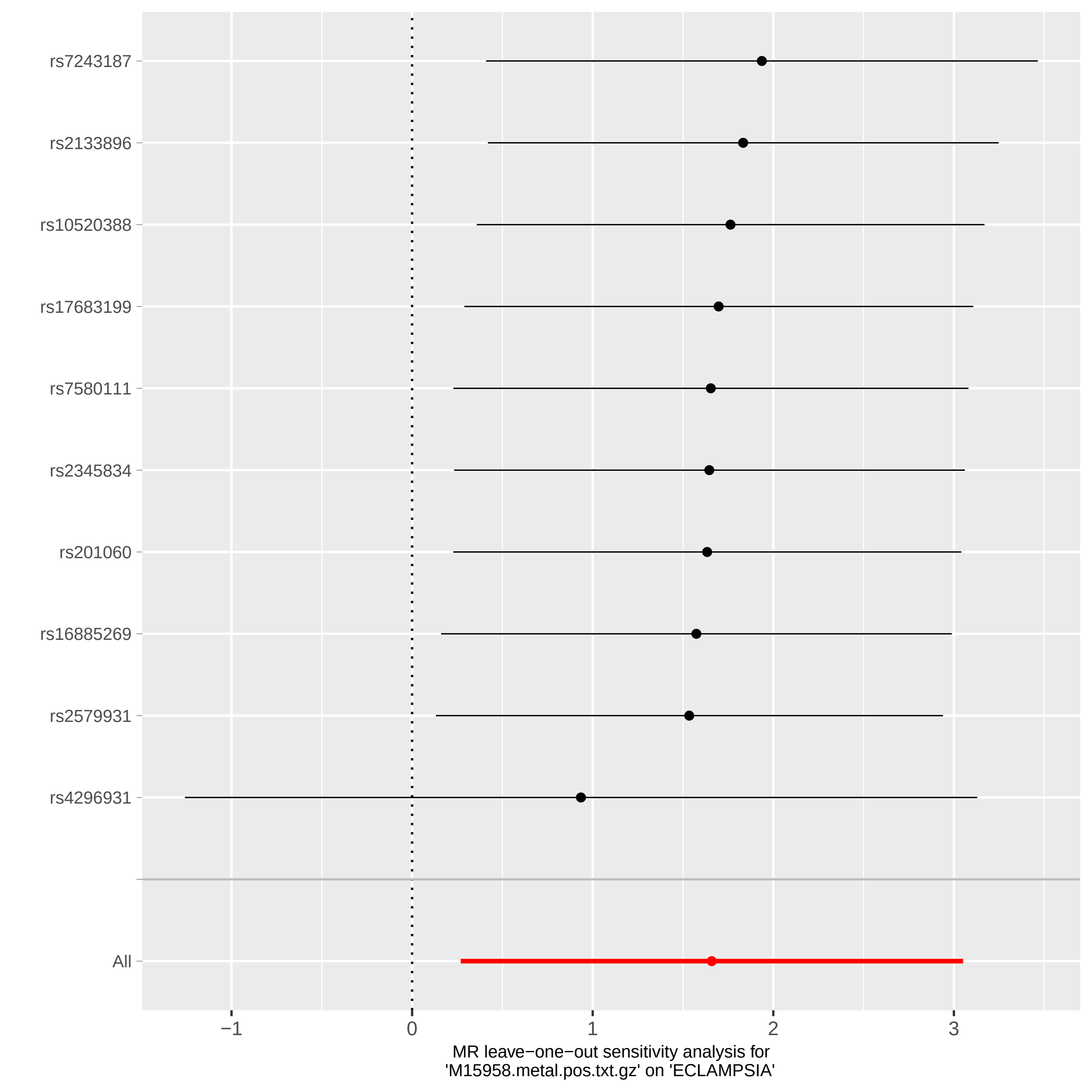

rs7243187
rs2133896
rs10520388
rs17683199
rs7580111
rs2345834
rs201060
rs16885269
rs2579931
rs4296931
All
−1
0
1
2
3
MR leave−one−out sensitivity analysis for
'M15958.metal.pos.txt.gz' on 'ECLAMPSIA'

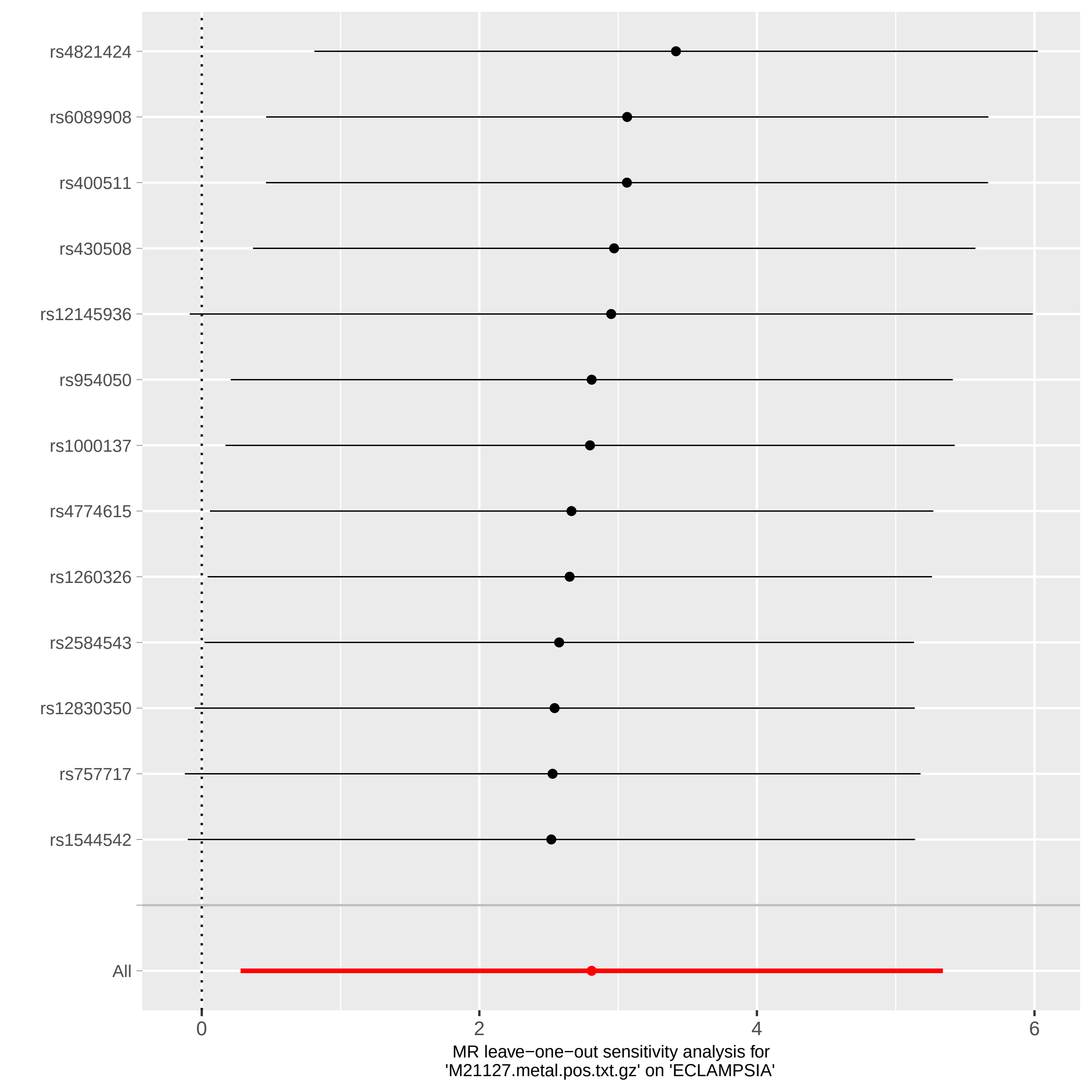
rs4821424
rs6089908
rs400511
rs430508
rs12145936
rs954050
rs1000137
rs4774615
rs1260326
rs2584543
rs12830350
rs757717
rs1544542
All
0
2
4
6
MR leave−one−out sensitivity analysis for
'M21127.metal.pos.txt.gz' on 'ECLAMPSIA'

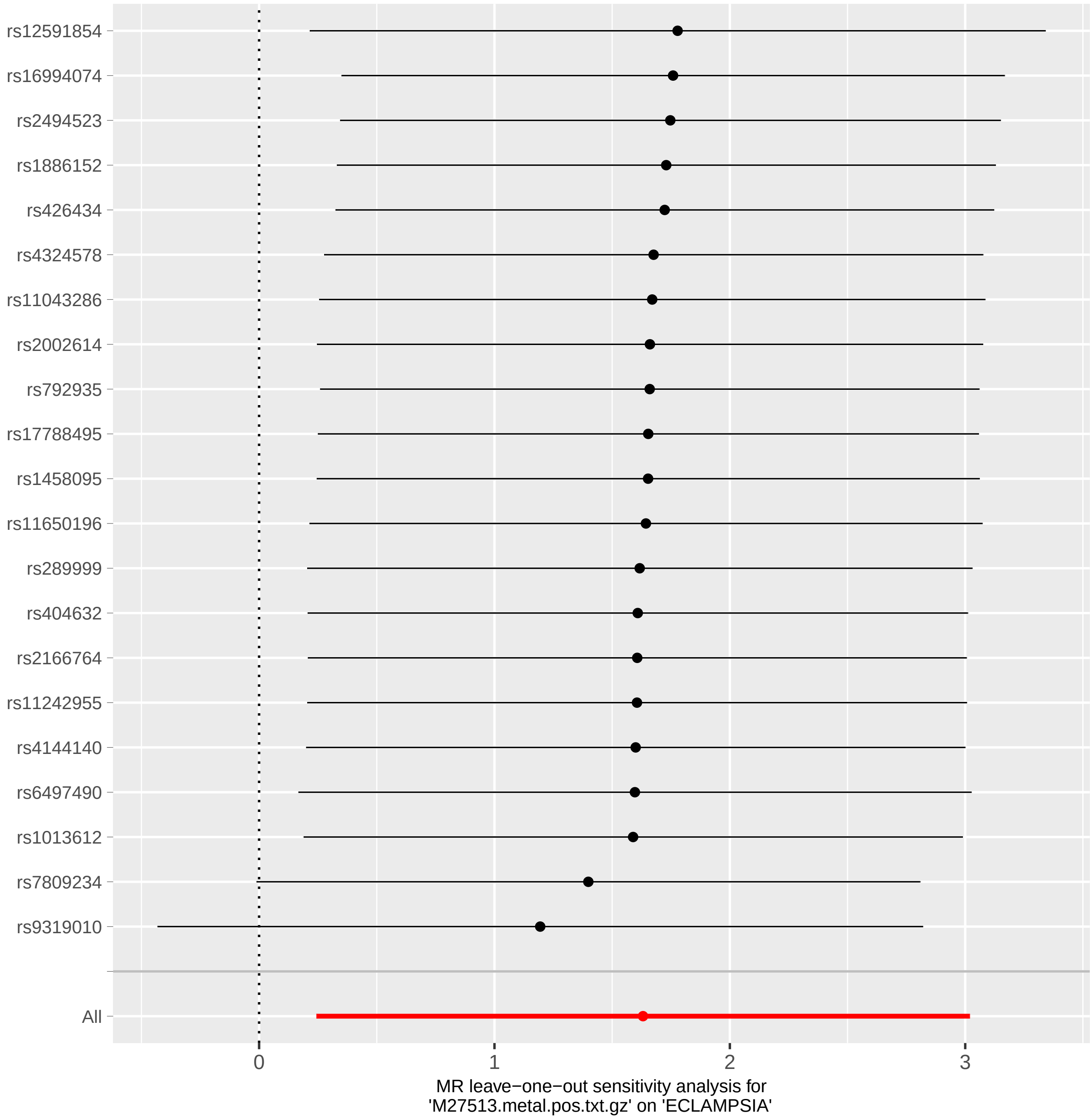
rs12591854
rs16994074
rs2494523
rs1886152
rs426434
rs4324578
rs11043286
rs2002614
rs792935
rs17788495
rs1458095
rs11650196
rs289999
rs404632
rs2166764
rs11242955
rs4144140
rs6497490
rs1013612
rs7809234
rs9319010
All
0
1
2
3
MR leave−one−out sensitivity analysis for
'M27513.metal.pos.txt.gz' on 'ECLAMPSIA'

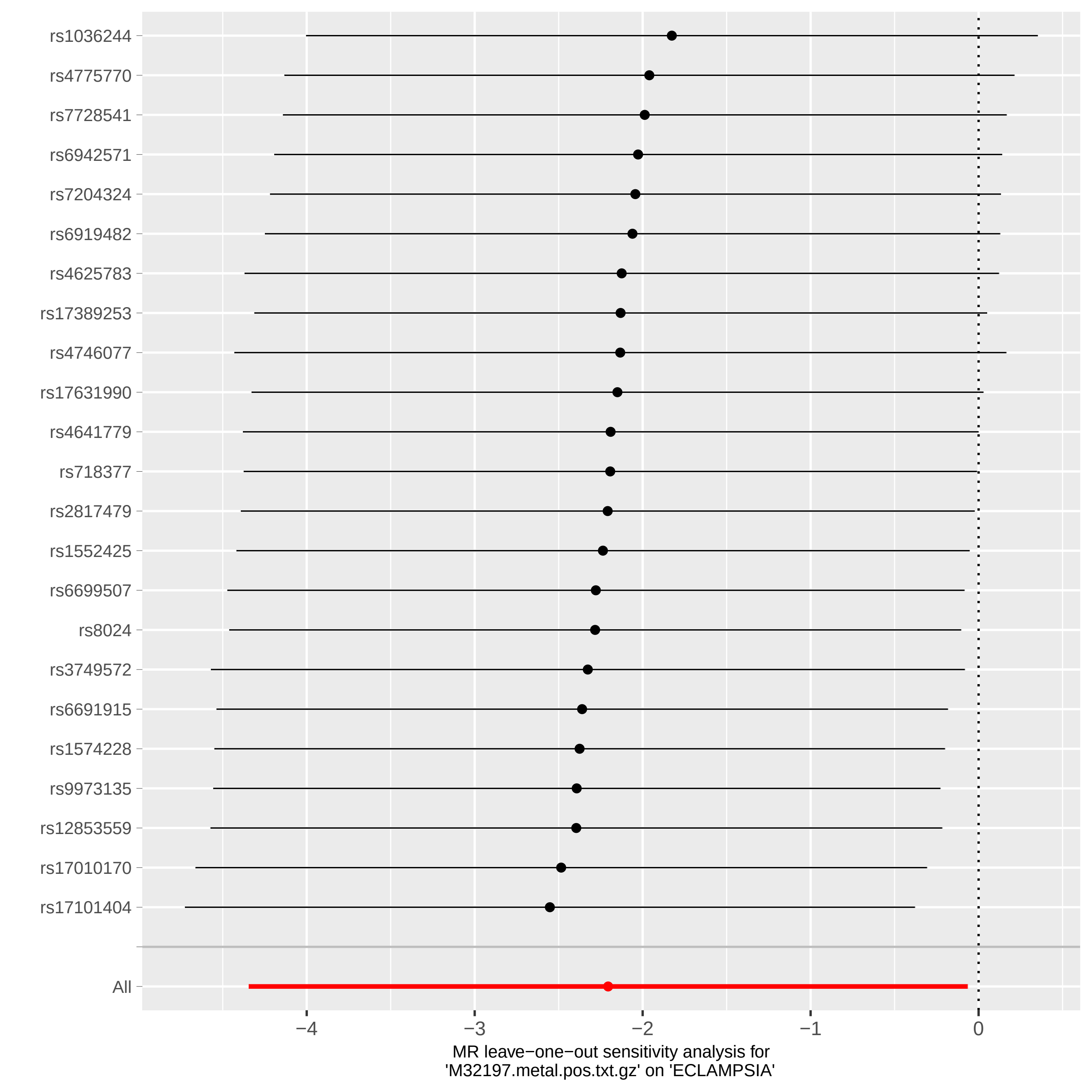

rs1036244
rs4775770
rs7728541
rs6942571
rs7204324
rs6919482
rs4625783
rs17389253
rs4746077
rs17631990
rs4641779
rs718377
rs2817479
rs1552425
rs6699507
rs8024
rs3749572
rs6691915
rs1574228
rs9973135
rs12853559
rs17010170
rs17101404
All
−4
−3
−2
−1
0
MR leave−one−out sensitivity analysis for
'M32197.metal.pos.txt.gz' on 'ECLAMPSIA'

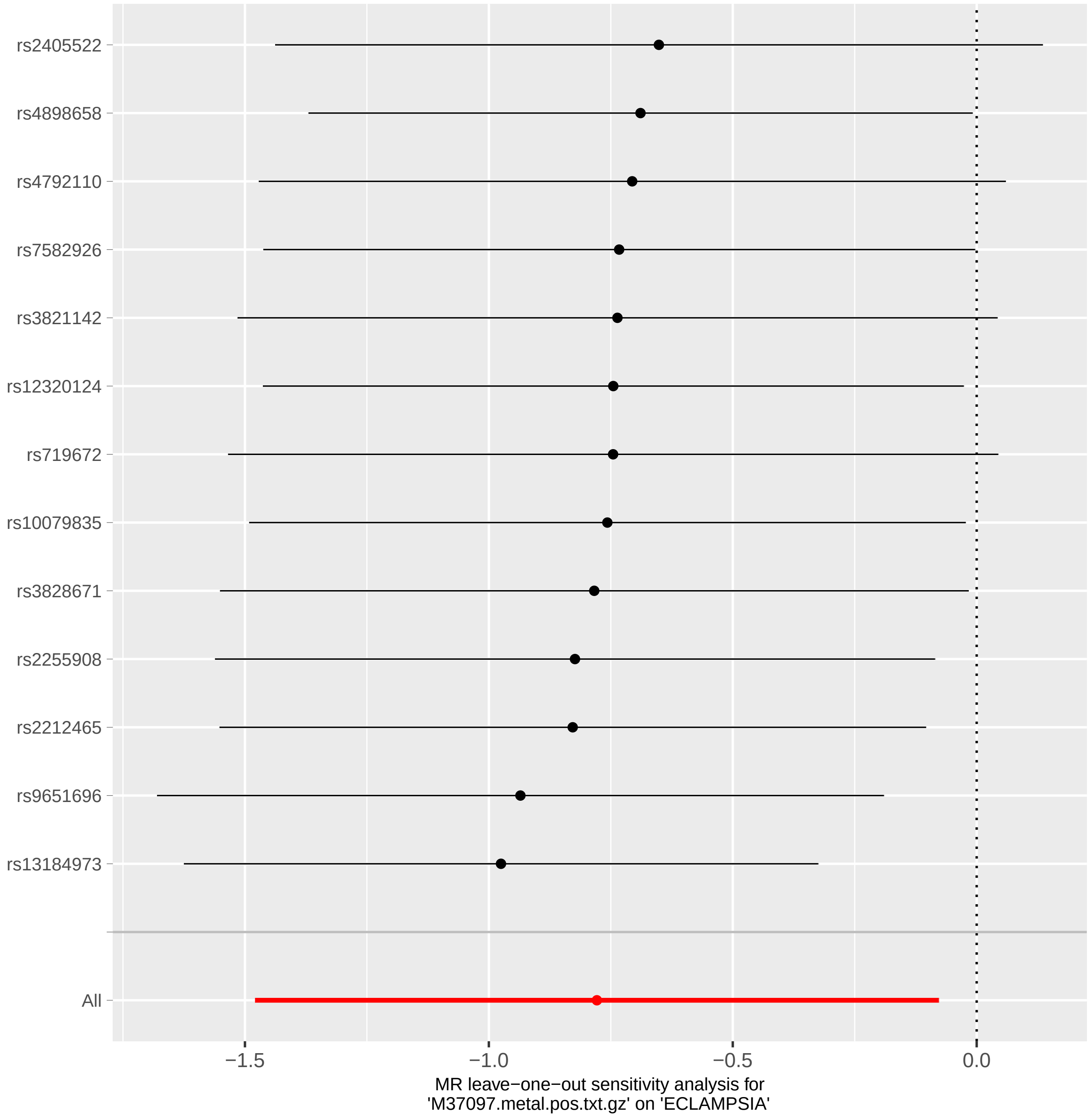
rs2405522
rs4898658
rs4792110
rs7582926
rs3821142
rs12320124
rs719672
rs10079835
rs3828671
rs2255908
rs2212465
rs9651696
rs13184973
All
−1.5
−1.0
−0.5
0.0
MR leave−one−out sensitivity analysis for
'M37097.metal.pos.txt.gz' on 'ECLAMPSIA'
