# Supplementary material for: The relationship between human blood metabolites and preeclampsia-eclampsia: A Mendelian randomization study
Source: Medicine (Baltimore). 2024 Mar 29;103(13):e37505. doi: 10.1097/MD.0000000000037505 (PMC10977518; doi:10.1097/MD.0000000000037505)
Supplement: Supplementary file 8 [file medi-103-e37505-s008.pdf]

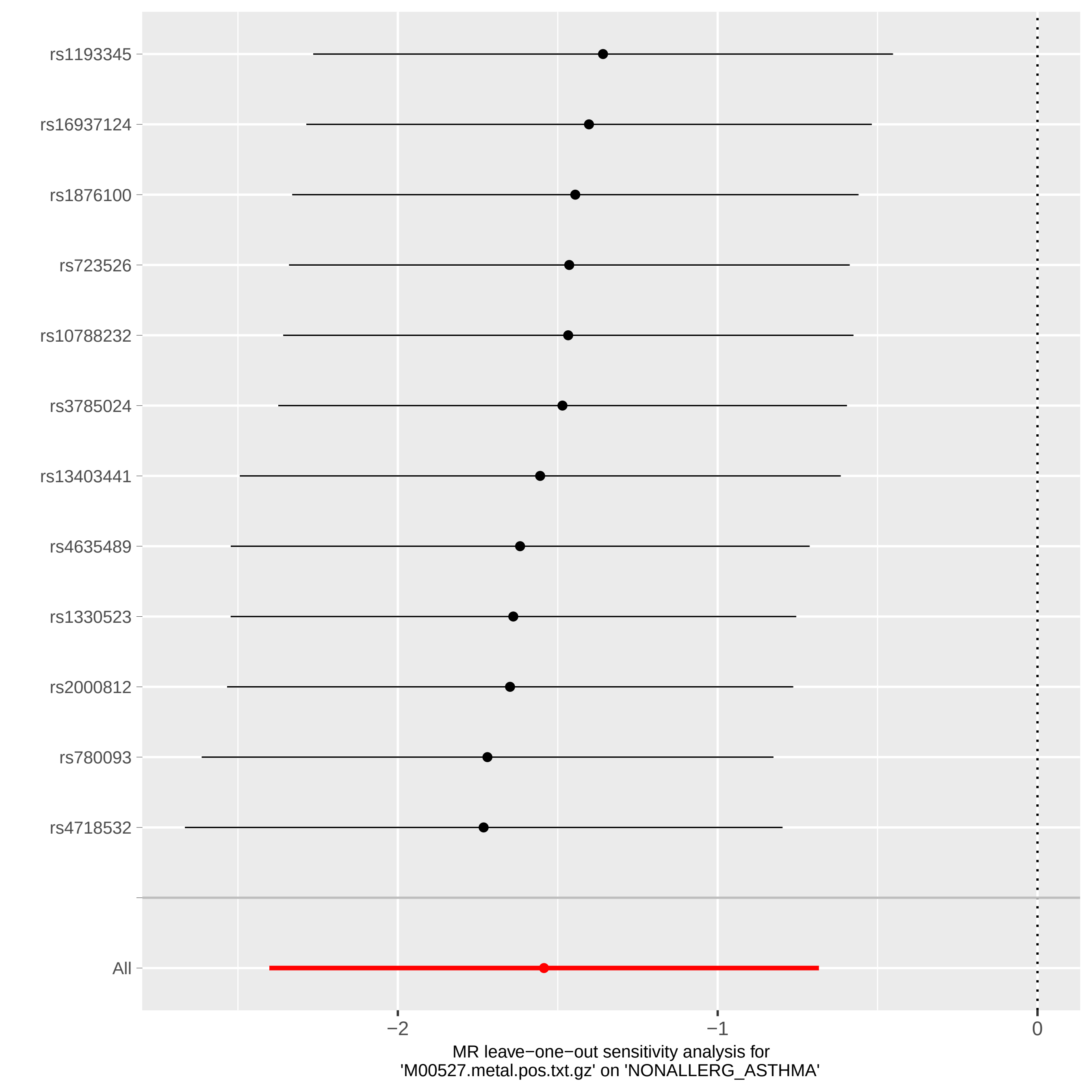

rs1193345
rs16937124
rs1876100
rs723526
rs10788232
rs3785024
rs13403441
rs4635489
rs1330523
rs2000812
rs780093
rs4718532
All
−2
−1
0
MR leave−one−out sensitivity analysis for
'M00527.metal.pos.txt.gz' on 'NONALLERG_ASTHMA'

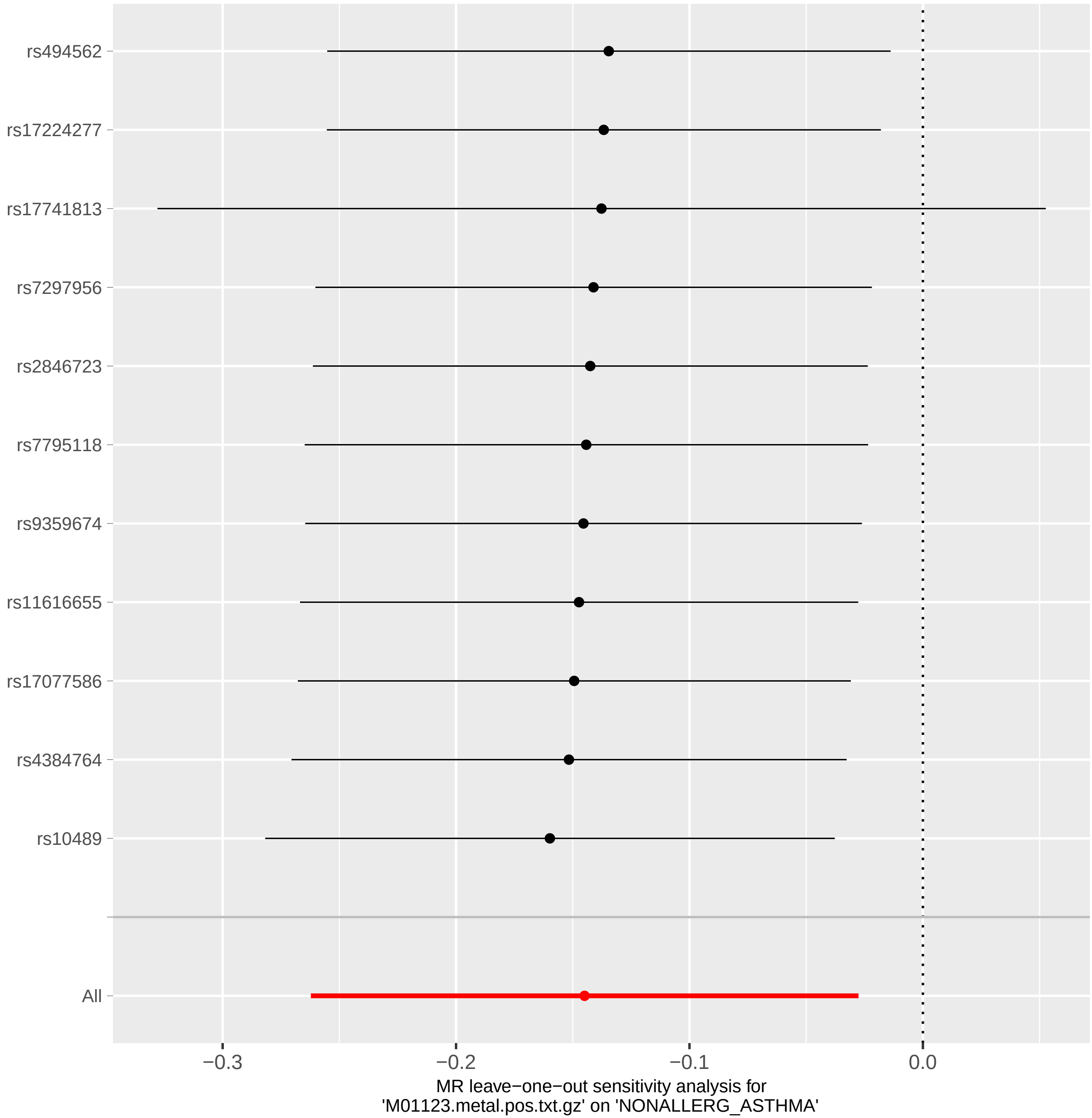

rs494562
rs17224277
rs17741813
rs7297956
rs2846723
rs7795118
rs9359674
rs11616655
rs17077586
rs4384764
rs10489
All
−0.3
−0.2
−0.1
0.0
MR leave−one−out sensitivity analysis for
'M01123.metal.pos.txt.gz' on 'NONALLERG_ASTHMA'

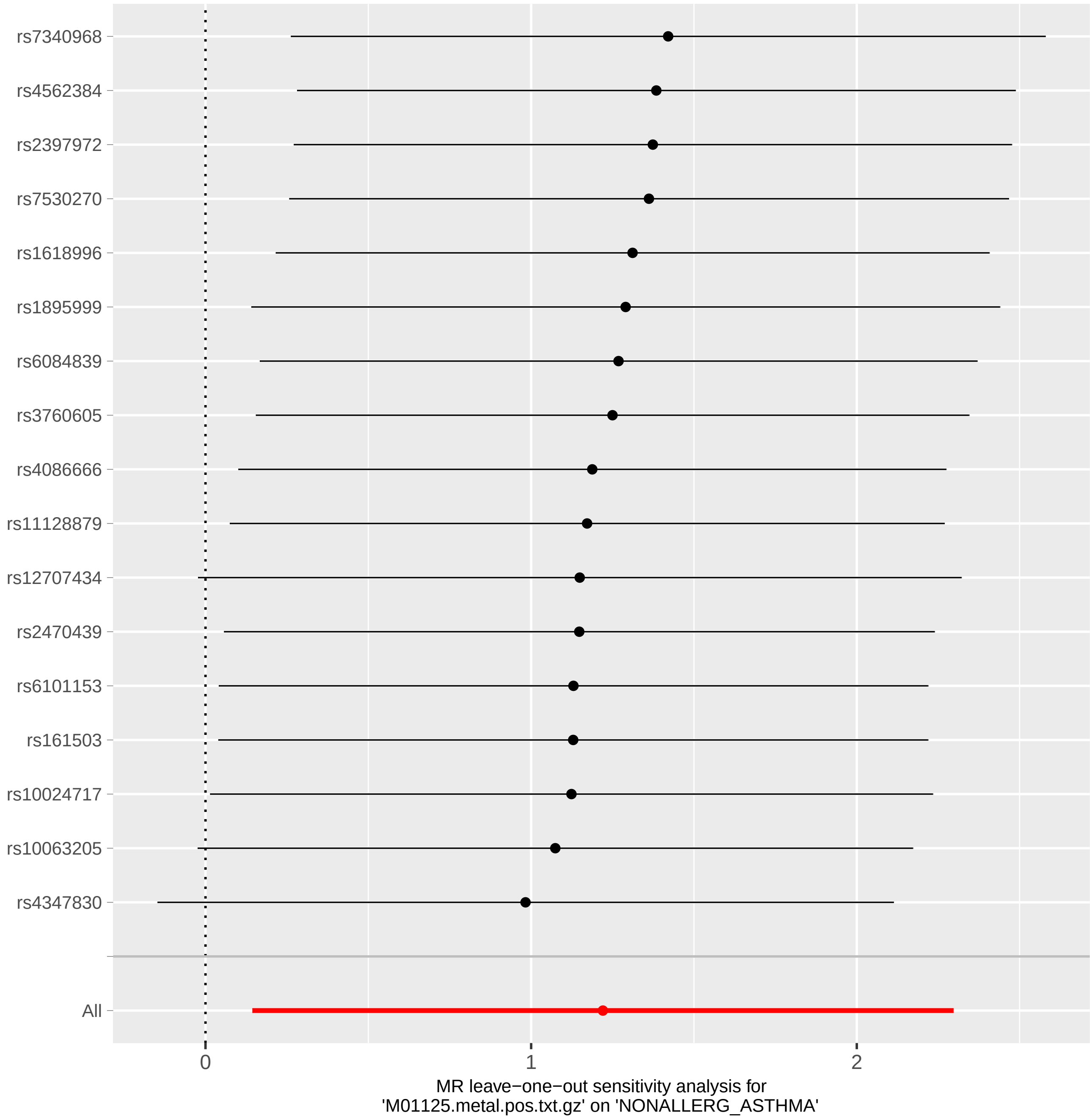

rs7340968
rs4562384
rs2397972
rs7530270
rs1618996
rs1895999
rs6084839
rs3760605
rs4086666
rs11128879
rs12707434
rs2470439
rs6101153
rs161503
rs10024717
rs10063205
rs4347830
All
0
1
2
MR leave−one−out sensitivity analysis for
'M01125.metal.pos.txt.gz' on 'NONALLERG_ASTHMA'

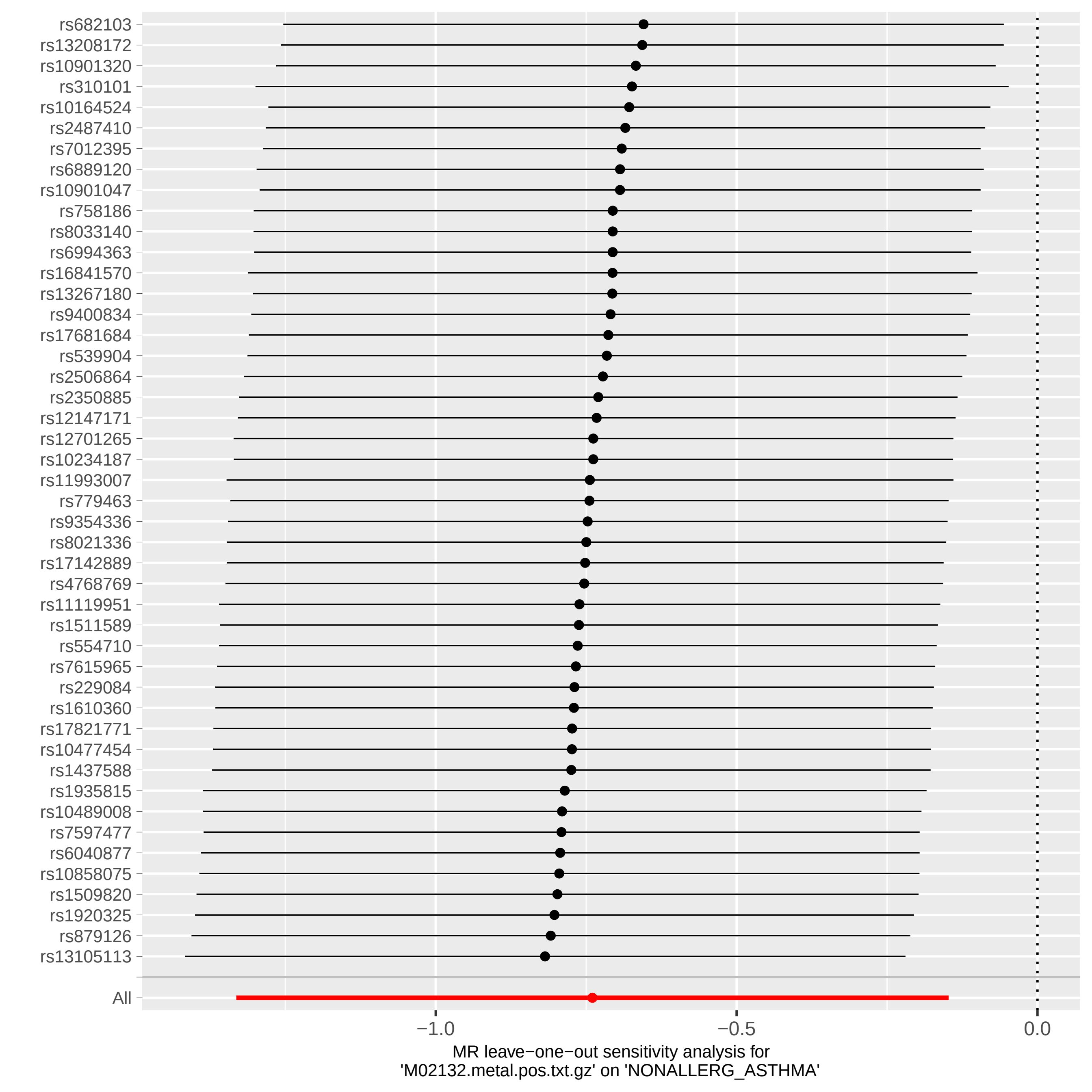
rs682103
rs13208172
rs10901320
rs310101
rs10164524
rs2487410
rs7012395
rs6889120
rs10901047
rs758186
rs8033140
rs6994363
rs16841570
rs13267180
rs9400834
rs17681684
rs539904
rs2506864
rs2350885
rs12147171
rs12701265
rs10234187
rs11993007
rs779463
rs9354336
rs8021336
rs17142889
rs4768769
rs11119951
rs1511589
rs554710
rs7615965
rs229084
rs1610360
rs17821771
rs10477454
rs1437588
rs1935815
rs10489008
rs7597477
rs6040877
rs10858075
rs1509820
rs1920325
rs879126
rs13105113
All
−1.0
−0.5
0.0
MR leave−one−out sensitivity analysis for
'M02132.metal.pos.txt.gz' on 'NONALLERG_ASTHMA'

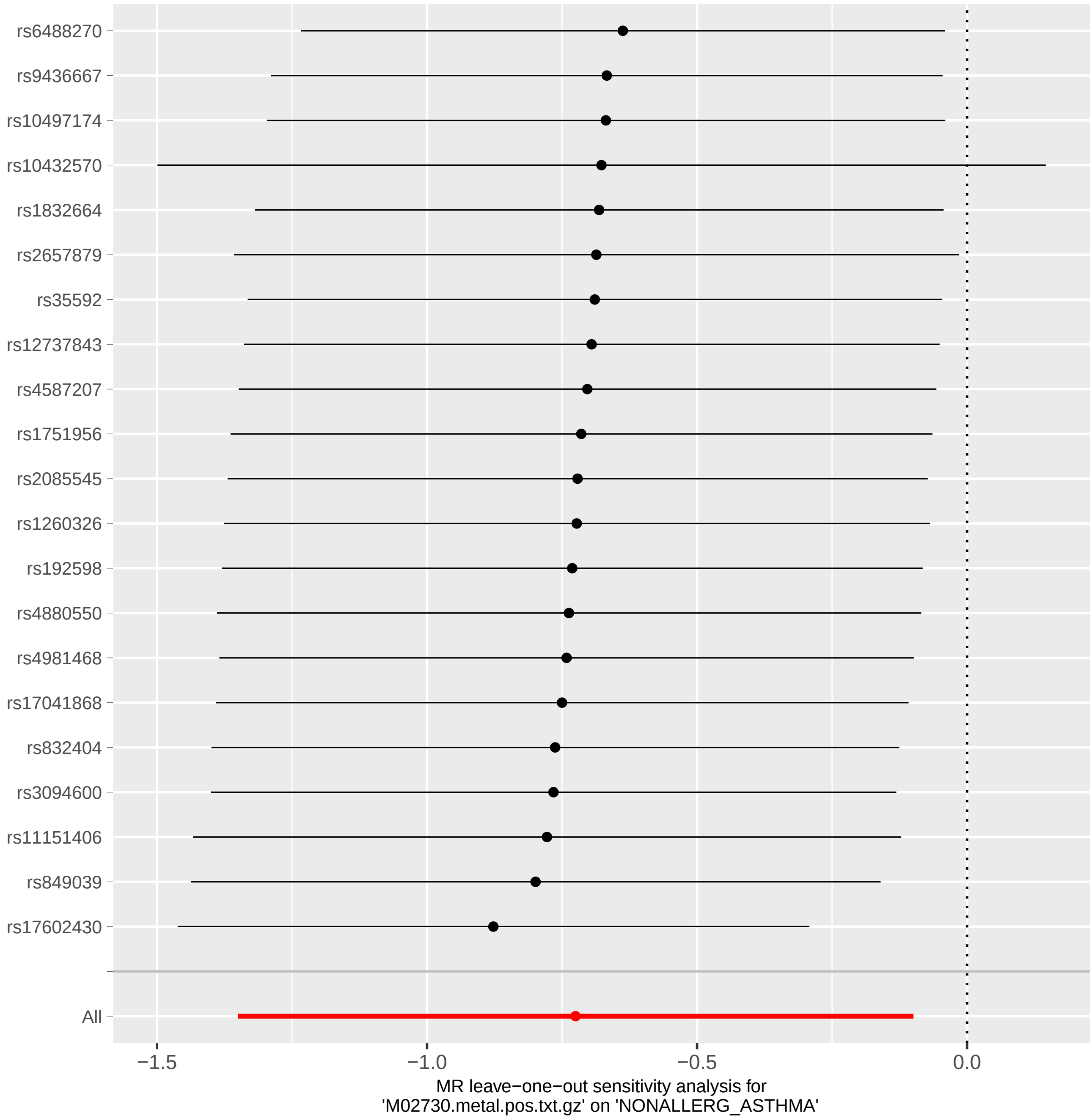
rs6488270
rs9436667
rs10497174
rs10432570
rs1832664
rs2657879
rs35592
rs12737843
rs4587207
rs1751956
rs2085545
rs1260326
rs192598
rs4880550
rs4981468
rs17041868
rs832404
rs3094600
rs11151406
rs849039
rs17602430
All
−1.5
−1.0
−0.5
0.0
MR leave−one−out sensitivity analysis for
'M02730.metal.pos.txt.gz' on 'NONALLERG_ASTHMA'

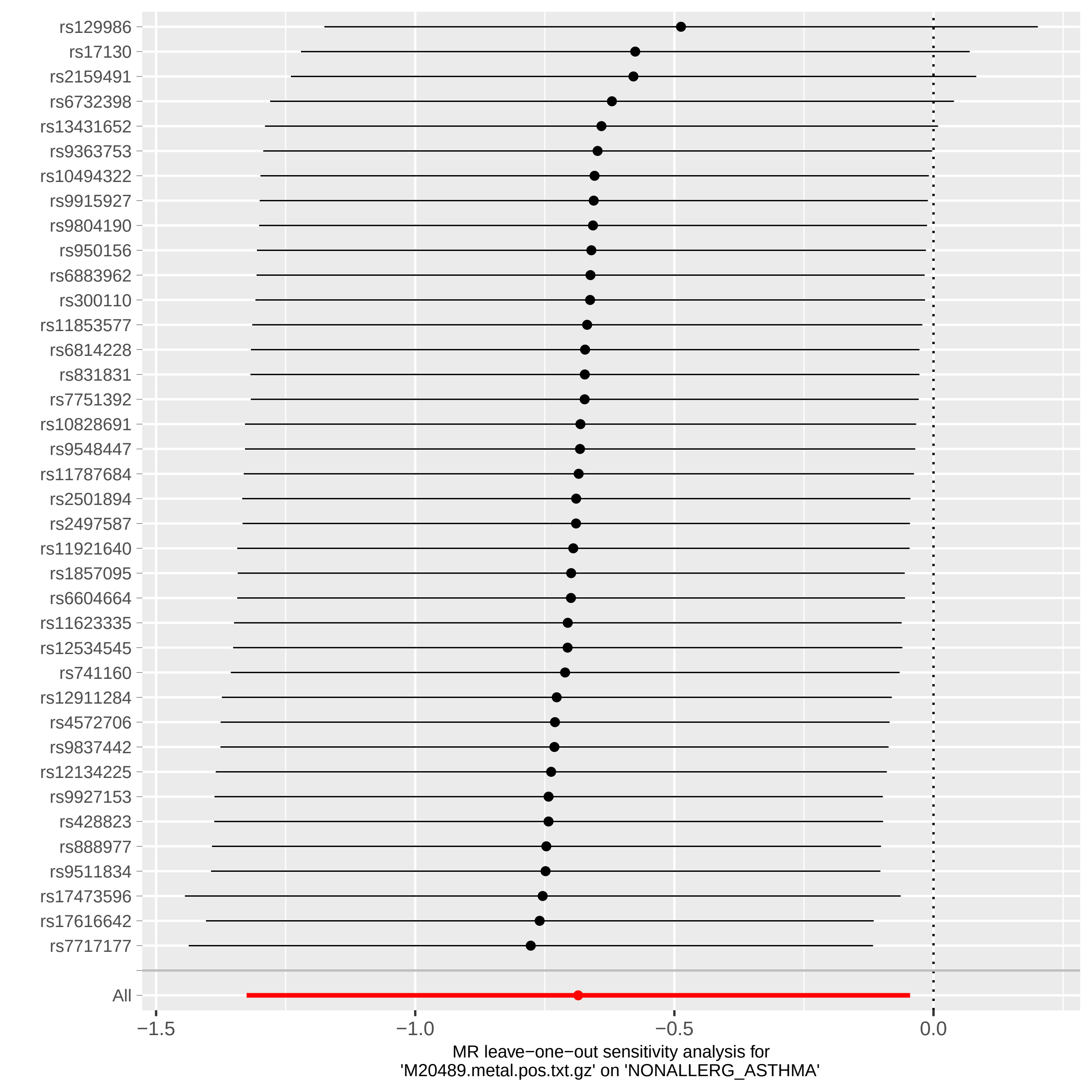
rs129986
rs17130
rs2159491
rs6732398
rs13431652
rs9363753
rs10494322
rs9915927
rs9804190
rs950156
rs6883962
rs300110
rs11853577
rs6814228
rs831831
rs7751392
rs10828691
rs9548447
rs11787684
rs2501894
rs2497587
rs11921640
rs1857095
rs6604664
rs11623335
rs12534545
rs741160
rs12911284
rs4572706
rs9837442
rs12134225
rs9927153
rs428823
rs888977
rs9511834
rs17473596
rs17616642
rs7717177
All
−1.5
−1.0
−0.5
0.0
MR leave−one−out sensitivity analysis for
'M20489.metal.pos.txt.gz' on 'NONALLERG_ASTHMA'

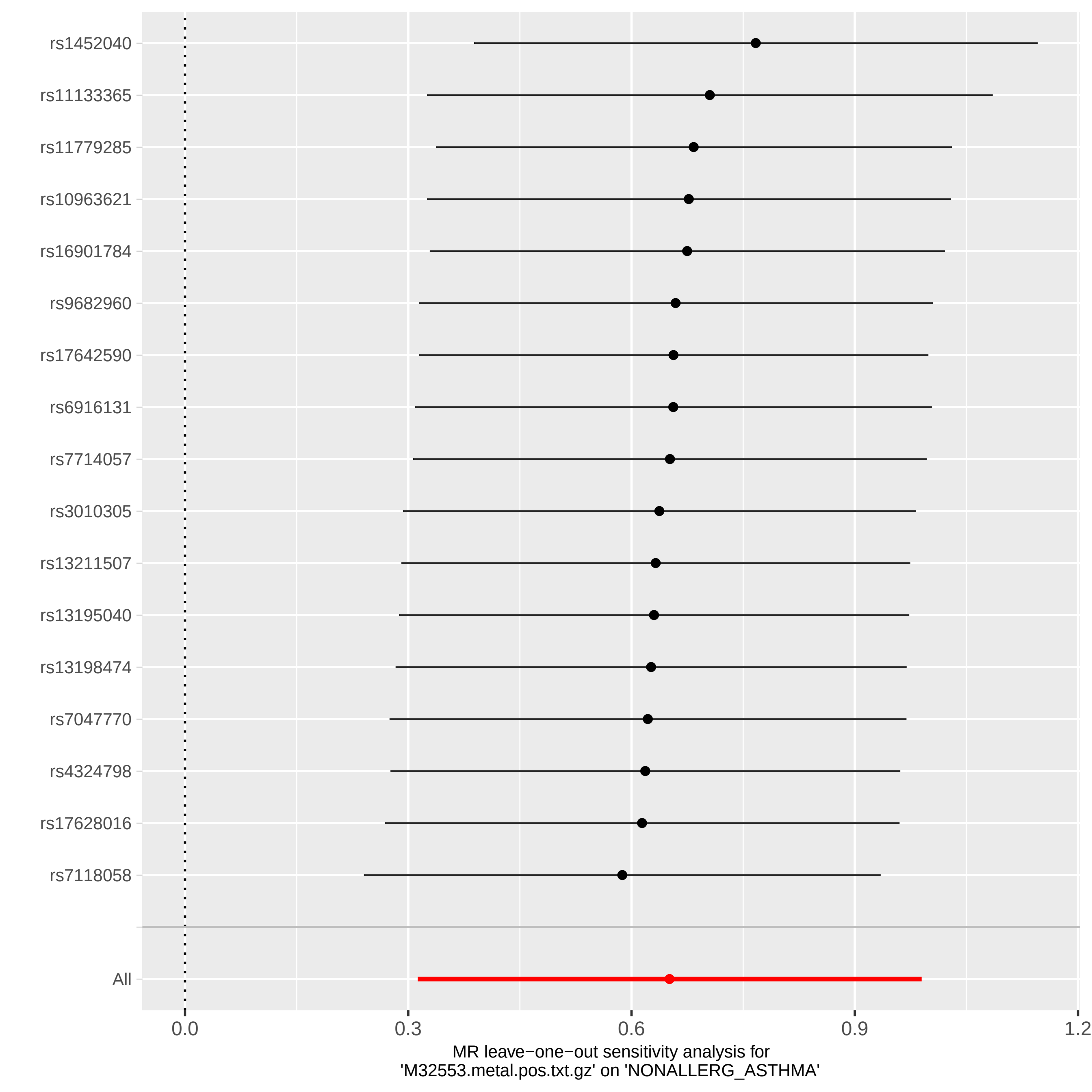
rs1452040
rs11133365
rs11779285
rs10963621
rs16901784
rs9682960
rs17642590
rs6916131
rs7714057
rs3010305
rs13211507
rs13195040
rs13198474
rs7047770
rs4324798
rs17628016
rs7118058
All
0.0
0.3
0.6
0.9
1.2
MR leave−one−out sensitivity analysis for
'M32553.metal.pos.txt.gz' on 'NONALLERG_ASTHMA'

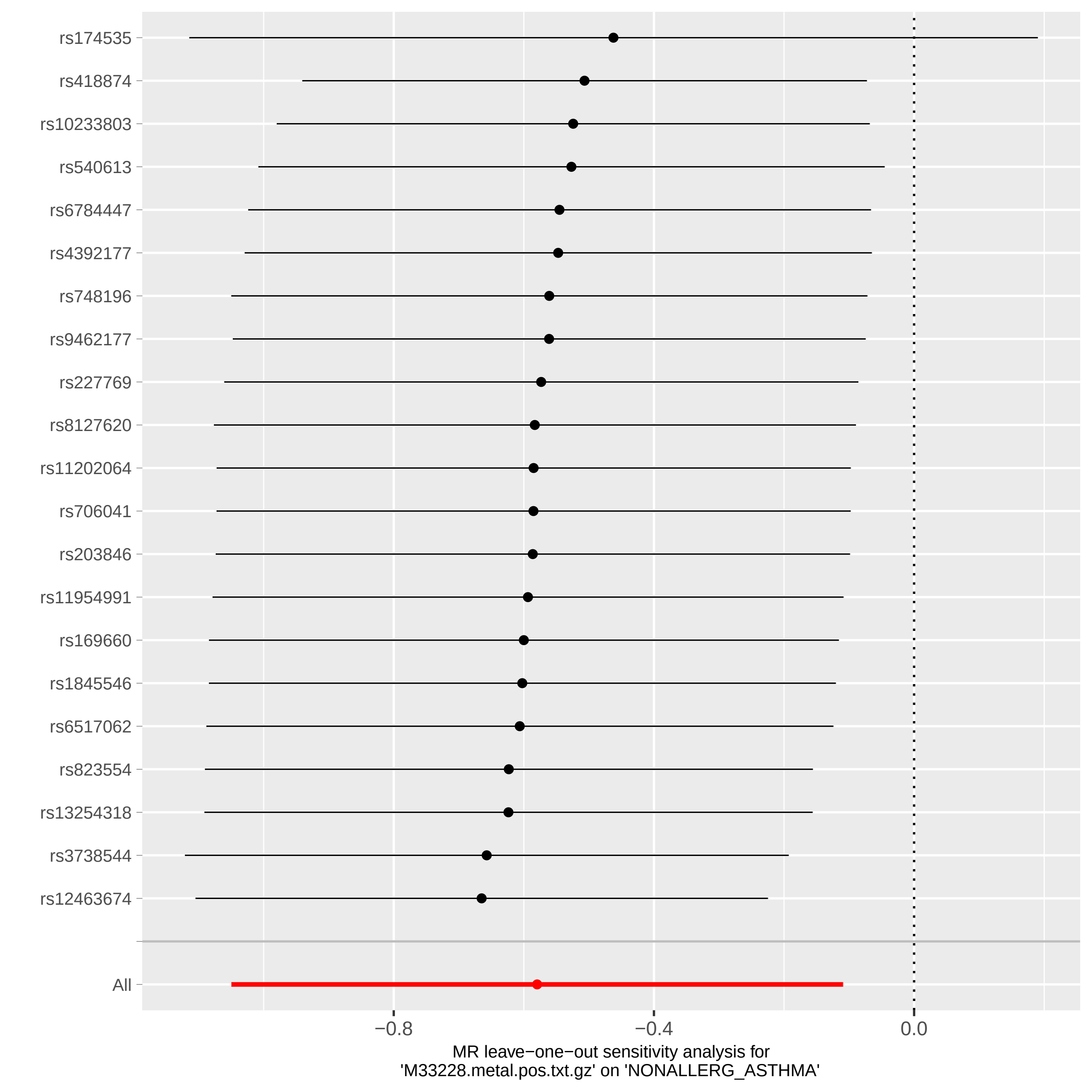

rs174535
rs418874
rs10233803
rs540613
rs6784447
rs4392177
rs748196
rs9462177
rs227769
rs8127620
rs11202064
rs706041
rs203846
rs11954991
rs169660
rs1845546
rs6517062
rs823554
rs13254318
rs3738544
rs12463674
All
−0.8
−0.4
0.0
MR leave−one−out sensitivity analysis for
'M33228.metal.pos.txt.gz' on 'NONALLERG_ASTHMA'

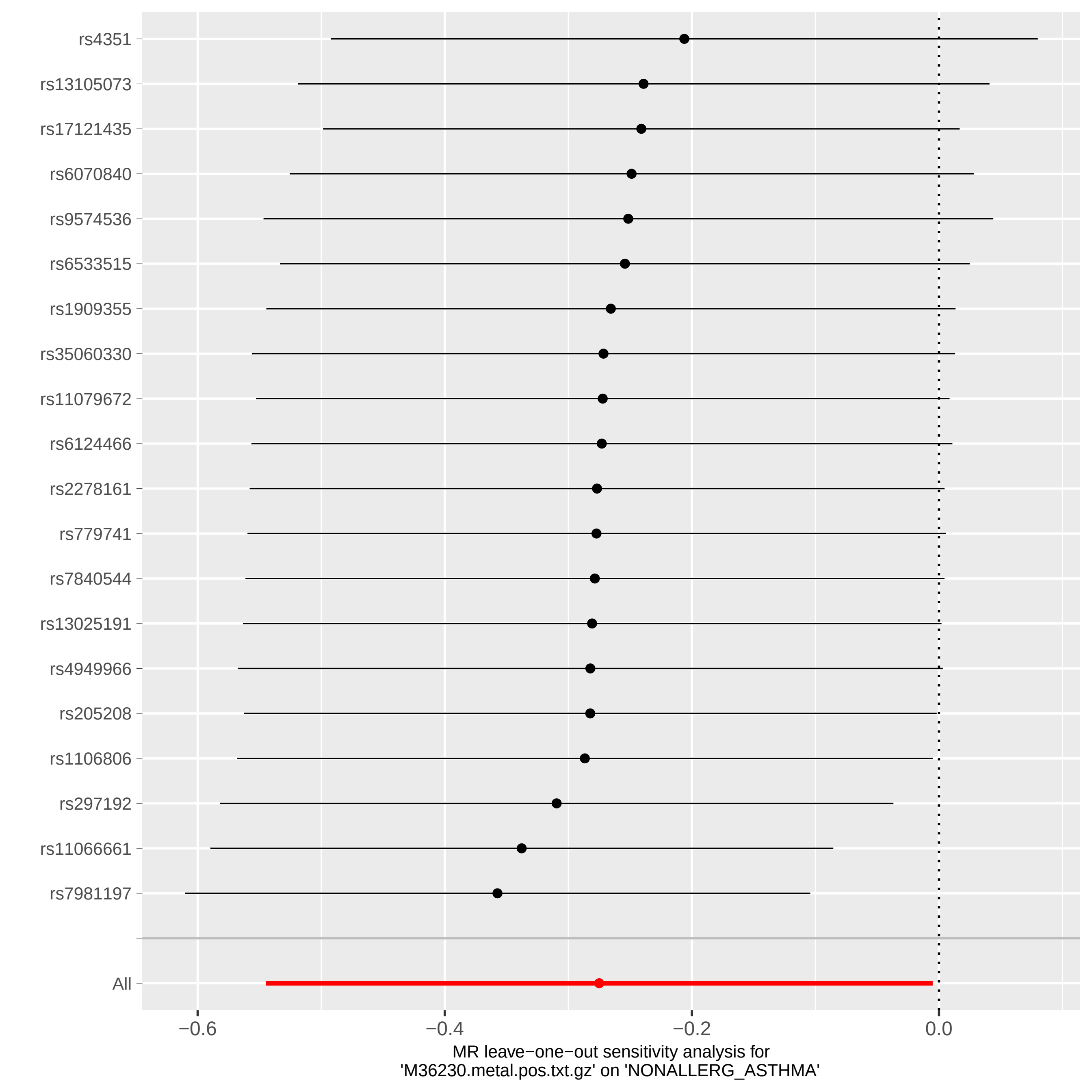

rs4351
rs13105073
rs17121435
rs6070840
rs9574536
rs6533515
rs1909355
rs35060330
rs11079672
rs6124466
rs2278161
rs779741
rs7840544
rs13025191
rs4949966
rs205208
rs1106806
rs297192
rs11066661
rs7981197
All
−0.6
−0.4
−0.2
0.0
MR leave−one−out sensitivity analysis for
'M36230.metal.pos.txt.gz' on 'NONALLERG_ASTHMA'

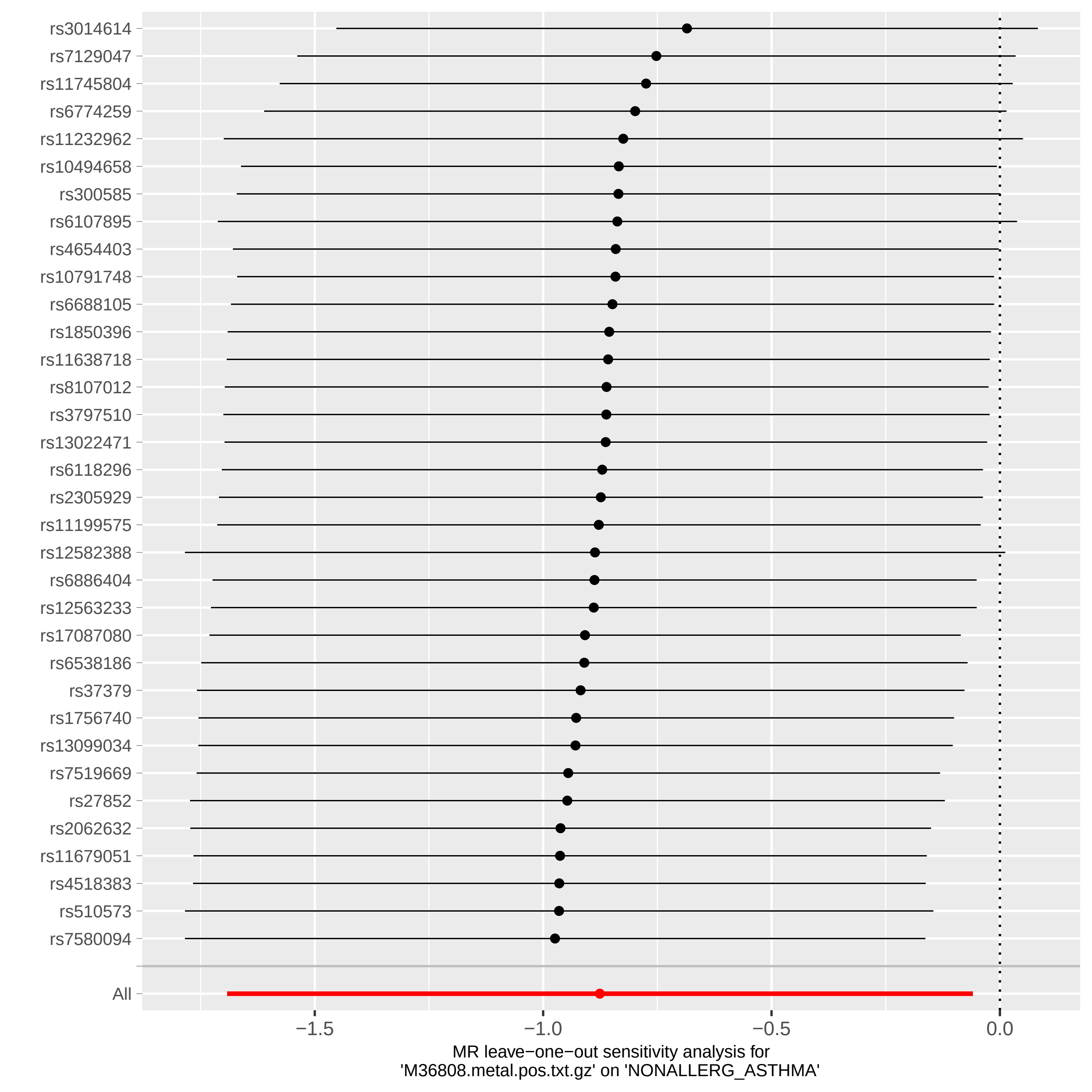
rs3014614
rs7129047
rs11745804
rs6774259
rs11232962
rs10494658
rs300585
rs6107895
rs4654403
rs10791748
rs6688105
rs1850396
rs11638718
rs8107012
rs3797510
rs13022471
rs6118296
rs2305929
rs11199575
rs12582388
rs6886404
rs12563233
rs17087080
rs6538186
rs37379
rs1756740
rs13099034
rs7519669
rs27852
rs2062632
rs11679051
rs4518383
rs510573
rs7580094
All
−1.5
−1.0
−0.5
0.0
MR leave−one−out sensitivity analysis for
'M36808.metal.pos.txt.gz' on 'NONALLERG_ASTHMA'
